# Comparative Genomics and Evolutionary Analysis of RNA-Binding Proteins of the CsrA Family in the Genus *Pseudomonas*

**DOI:** 10.3389/fmolb.2020.00127

**Published:** 2020-07-10

**Authors:** Patricio Martín Sobrero, Claudio Valverde

**Affiliations:** Laboratorio de Fisiología y Genética de Bacterias Beneficiosas para Plantas, Centro de Bioquímica y Microbiología del Suelo, Departamento de Ciencia y Tecnología, Universidad Nacional de Quilmes - CONICET, Buenos Aires, Argentina

**Keywords:** RNA-binding proteins, CsrA, RsmA, *Pseudomonas*, comparative genomics, evolutionary analysis, plasmids, phages

## Abstract

Gene expression is adjusted according to cellular needs through a combination of mechanisms acting at different layers of the flow of genetic information. At the posttranscriptional level, RNA-binding proteins are key factors controlling the fate of nascent and mature mRNAs. Among them, the members of the CsrA family are small dimeric proteins with heterogeneous distribution across the bacterial tree of life, that act as global regulators of gene expression because they recognize characteristic sequence/structural motifs (short hairpins with GGA triplets in the loop) present in hundreds of mRNAs. The regulatory output of CsrA binding to mRNAs is counteracted in most cases by molecular mimic, non-protein coding RNAs that titrate the CsrA dimers away from the target mRNAs. In γ-proteobacteria, the regulatory modules composed by CsrA homologs and the corresponding antagonistic sRNAs, are mastered by two-component systems of the GacS-GacA type, which control the transcription and the abundance of the sRNAs, thus constituting the rather linear cascade Gac-Rsm that responds to environmental or cellular signals to adjust and coordinate the expression of a set of target genes posttranscriptionally. Within the γ-proteobacteria, the genus *Pseudomonas* has been shown to contain species with different number of active CsrA (RsmA) homologs and of molecular mimic sRNAs. Here, with the help of the increasing availability of genomic data we provide a comprehensive state-of-the-art picture of the remarkable multiplicity of CsrA lineages, including novel yet uncharacterized paralogues, and discuss evolutionary aspects of the CsrA subfamilies of the genus *Pseudomonas*, and implications of the striking presence of *csrA* alleles in natural mobile genetic elements (phages and plasmids).

## Introduction

Regulation of gene expression is key to the metabolic economy of the prokaryotic cell. The pathway from the gene sequence to the encoded final active polypeptide offers several opportunities for adjusting the flow of gene expression. For decades, the focus of gene regulatory processes in prokaryotes has been the control of transcription initiation by protein regulatory factors (i.e., transcriptional regulation), and it was deemed a taxonomically widespread and most efficient way to limit the amount of macromolecule synthesis depending on cues perceived from the environment or the inner cell compartment. However, during the last 30 years there has been an enormous input of genetic, biochemical, physiological, and omics data strongly supporting the pervasive and critical role of genetic regulatory mechanisms that operate on top of transcription initiation to modulate the fate of the nascent or mature transcripts (i.e., posttranscriptional control of gene expression). Although in most cases the degree of regulatory effect introduced by these mechanisms is mild and serve to fine-tune the outcome of transcriptional regulatory controls, in some cases, posttranscriptional regulations can introduce a quantitatively significant adjustment to become the master control of the genetic flow of a certain pathway or process. At the molecular level, the posttranscriptional control of gene expression can be executed by sequence portions of the mRNA themselves (e.g., cis-acting motifs like riboswitches and thermosensors), by non-protein coding, small regulatory RNAs (sRNAs) that base-pair with mRNAs, or by RNA-binding proteins that have preference for sequence and/or structural motifs on target mRNAs. For updates on *cis*-acting RNA regulatory elements and sRNAs we refer the reader to recent comprehensive reviews (Quereda and Cossart, 2017; Desgranges et al., 2019; Bedard et al., 2020; Jorgensen et al., 2020; Mandin and Johansson, 2020).

Prokaryotic genomes encode over a hundred of RNA-binding proteins, being the majority of them devoted to scaffold the ribosomal subunits or to catalytically process RNA molecules for maturation or defense (Holmqvist and Vogel, 2018). A third functional class of RNA-binding proteins is involved in posttranscriptional control of gene expression (Quendera et al., 2020), with some outstanding cases acting as global regulators of major influence in the fate of hundreds of mRNAs, as is the case of the broadly studied chaperone Hfq (Vogel and Luisi, 2011; Sobrero and Valverde, 2012; Kavita et al., 2018; Santiago-Frangos and Woodson, 2018), or the members of the CsrA family (Romeo and Babitzke, 2019), which is the subject of this article. Here, we will review the features of the RNA-binding proteins of the CsrA superfamily, with an emphasis on the representatives of the genus *Pseudomonas*, for which our comparative genome analysis revealed a prolific evolutionary spreading of multiple paralogues.

## The CsrA Protein Family

**Figure 1:**
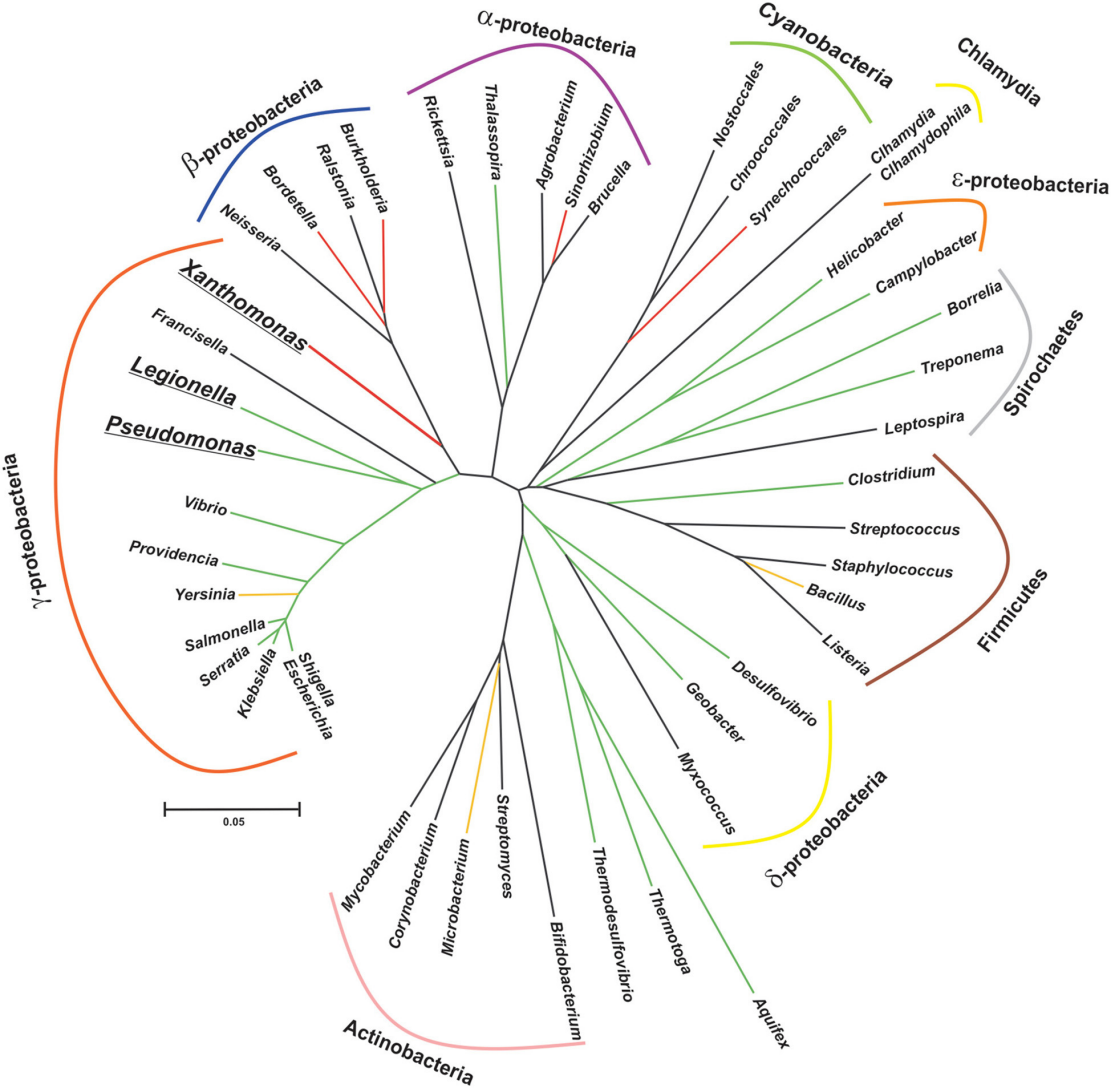
Patched phylogenetic distribution of genes encoding CsrA homologs in major eubacterial taxa. The phylogenetic tree was generated on the basis of the 16S rDNA sequences of one representative species for each eubacterial genus present in the diagram. The evolutionary history was inferred using the Neighbor-Joining method (Saitou and Nei, 1987). The optimal tree with the sum of branch length = 3.385 is shown. The tree is drawn to scale, with branch lengths in the same units as those of the evolutionary distances used to infer the phylogenetic tree. The evolutionary distances were computed using the Kimura 2-parameter method (Kimura, 1980) and are in the units of the number of base substitutions per site. This analysis involved 46 nucleotide sequences. All positions with <95% site coverage were eliminated, i.e., fewer than 5% alignment gaps, missing data, and ambiguous bases were allowed at any position (partial deletion option). There were a total of 1,174 positions in the final dataset. Evolutionary analyses were conducted in MEGA X (Kumar et al., 2018). The reference bar length indicates the number of nucleotide substitution per site. The branch color corresponds to the proportion of species per class that contain at least one *csrA* allele, as follows: black, 0% (no homologs in this class); red, <10%; yellow, >50%; green, 100%. Bolded and underlined taxon names denote those genera which present more than one allele per genome.

CsrA stands for Carbon storage regulator A and it was discovered almost 30 years ago in a Tn*5* mutagenic screen of *E. coli* as a 61-amino acid polypeptidic regulatory factor of glycogen biosynthesis genes, and soon revealed its role as a global regulator of gene expression (Romeo et al., 1993). The regulatory mechanism underlying CsrA activity was obscure at that time. The first study to explore the phylogenetic distribution relied on the detection of homolog sequences by Southern blot using a PCR probe consisting of the *E. coli csrA* gene, and a sequence homology search in nucleotide sequence databases (White et al., 1996); although a very limited number of bacterial genomes were explored with both techniques, the results suggested a broad distribution of this kind of novel regulatory protein in eubacteria. Currently, the Pfam entry CsrA (PF02599) and the InterPro entry IPR003751, together include over 16.000 polypeptidic sequences from more than 2900 species. Intriguingly, representatives of this large protein superfamily have been detected exclusively in the chromosomes of eubacterial species (Figure 1). Nevertheless, the increasing availability of genomes from environmental metagenomic projects may prompt the identification of CsrA remote homologs in archaeal lineages.

**Figure 2:**
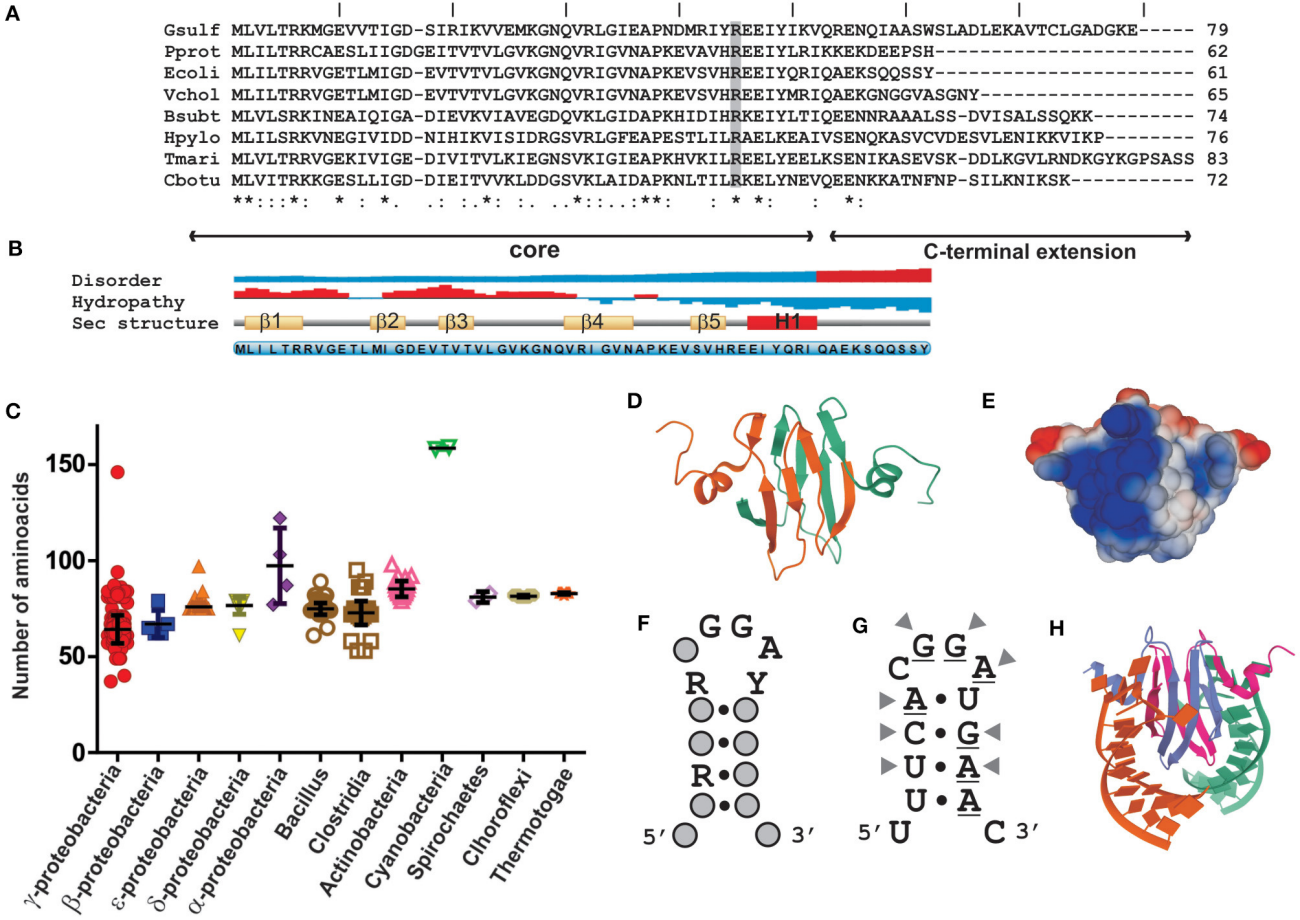
Structural properties of CsrA proteins and their interaction with RNA sequence/structural motifs. **(A)** Primary sequence alignment of CsrA polypeptides from selected eubacterial species: Gsulf, *Geobacter sulfurreducens*; Pprot, *Pseudomonas protegens*; Ecoli, *Escherichia coli*; Vchol, *Vibrio cholerae*; Bsubt, *Bacillus subtilis*; Hpylo, *Helicobacter pylori*; Tmari, *Thermotoga maritima*; Cbotu, *Clostridium botulinum*. The degree of residue conservation is indicated below each position of the alignment, as follows: an (*) indicates positions with a fully conserved residue; a (:) indicates conservation between amino acid groups of strongly similar biochemical properties; a (.) indicates conservation between groups of weakly similar properties. The residue shaded in gray (R) corresponds to the conserved arginine (R44 in *E. coli* CsrA) that is essential for the RNA-binding activity of CsrA proteins (Heeb et al., 2006; Schubert et al., 2007). The core region comprises the prototypical portion of the molecule that defines the CsrA fold, namely the β1β2β3β4β5H1 arrangement; the C-terminal extension is highly variable among different taxa. **(B)** Secondary structure properties the *E. coli* CsrA polypeptide. The data was generated with the Protein feature view tool of the RCSB PDB server www.rcsb.org (Berman et al., 2000). **(C)** The graph shows the length distribution of CsrA homologs within different eubacterial classes. For each group the average length (nr. of residues) ± standard deviation is indicated. **(D)** Ribbon diagram of the solution quaternary structure of the *E. coli* CsrA dimeric protein (PDB 1Y00). **(E)** Predicted charge distribution along the surface of the dimeric *P. protegens* RsmE protein (PDB 2JPP). **(F)** Consensus sequence/structural motif preferentially bound by *E. coli* CsrA *in vivo*, as detected following CLIP-Seq (adapted from Potts et al., 2019). R = G or A, Y = C or U. **(G)** Predicted secondary structure of the 14-mer encompassing the Shine-Dalgarno sequence (underlined nucleotides) of the *hcnA* 5′-UTR from *P. protegens* strain CHA0, an experimentally validated target for the CsrA homolog protein RsmE (Lapouge et al., 2007; Schubert et al., 2007). The arrowheads point to the nucleotides contacted by specific RsmE residues in the complex shown in **(H)** (Schubert et al., 2007). **(H)** Ribbon diagram of the NMR solution structure of the RsmE–*hcnA* 5′-UTR complex (PDB 2JPP). Each RNA binding site of the dimeric RsmE protein is interacting with one of two identical synthetic 20-mers containing the sequence-structural motif shown in **(G)**.

Members of the CsrA family are rather well conserved polypeptides of relatively short length (65–75 residues on average) that function as homodimers (Figure 2). Heterodimerization in bacteria encoding more than one paralogue has not been demonstrated yet, but it may be plausible. The secondary structure predicted for most representatives indicate that CsrA monomers fold into five consecutive, antiparallel β-strands (β1β2β3β4β5) followed by one short α-helix (H1), and the unstructured C-terminus of variable length (Gutierrez et al., 2005) (Figure 2B). The dimeric and biologically active structure (Gutierrez et al., 2005; Rife et al., 2005; Heeb et al., 2006; Schubert et al., 2007) is formed by intertwining of β1 and β5, which results in a sandwich of two, five-stranded, antiparallel β-sheets, with the two α-helixes projected out from the dimer core (Figure 2D). The RNA-binding sites lay in the conserved and positively charged regions adjacent to strands β1, β4, β5, and the N-terminal region of helix H1, on each side of the dimer (Figure 2E). These two sites have a marked preference for RNA sequence/structural motifs characterized by short stem-loops exposing the trinucleotide GGA in the apical loop (Figure 2F). A strongly conserved arginine residue at the interface of β5 and H1 is fundamental for the recognition of the first G of the GGA trinucleotide, and its replacement abolishes the regulatory binding of CsrA proteins to their RNA targets (Heeb et al., 2006; Schubert et al., 2007).

**Figure 3:**
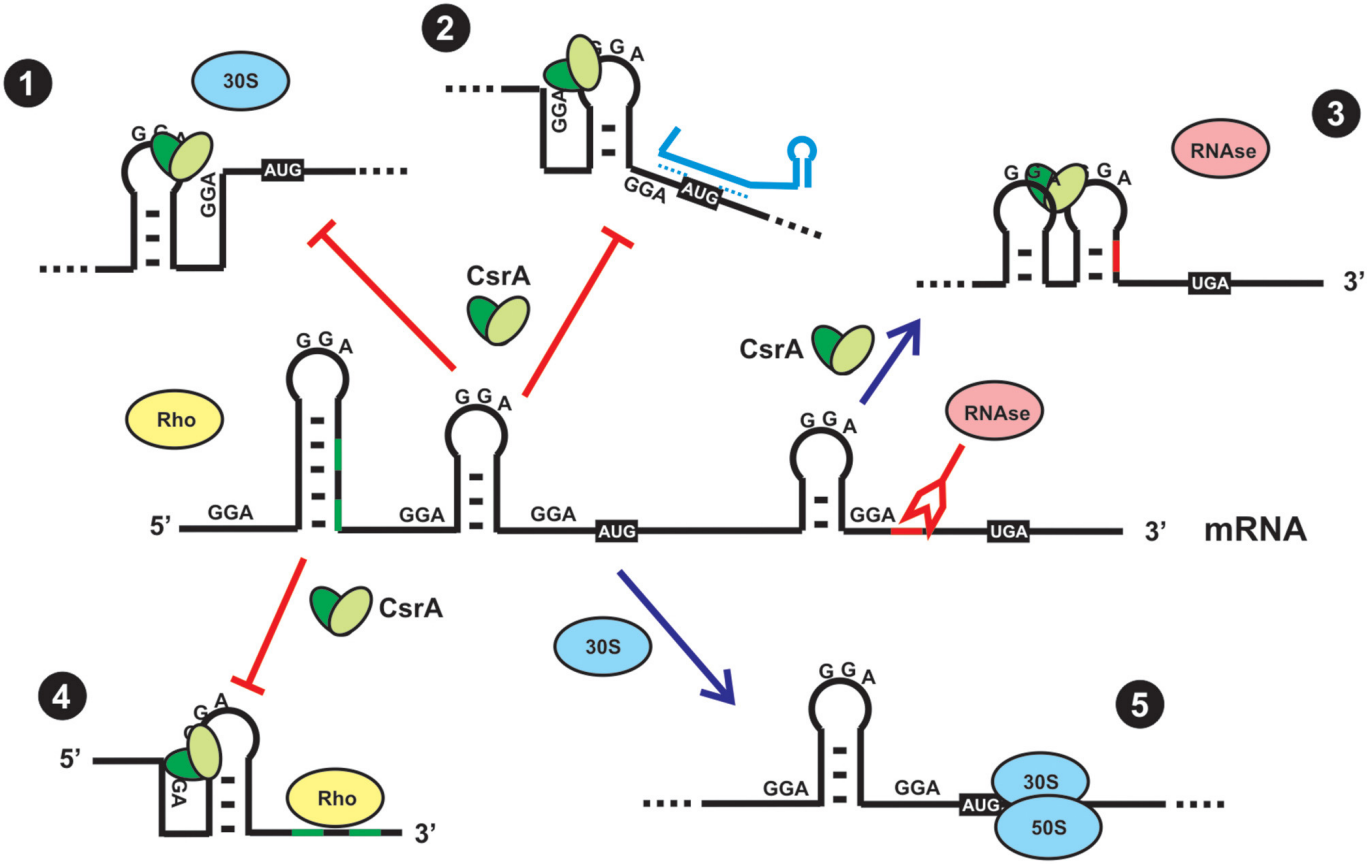
Direct and indirect regulatory effects of CsrA binding to a mRNA. (1) Direct translational repression. In most cases, CsrA binds to target sites around or within the Shine-Dalgarno region, and as a consequence it outcompetes binding of the small ribosomal subunit and its engagement in translation initiation. (2) Indirect translational repression. CsrA binds to target sites in the 5′-UTR and induces a conformational change that exposes the Shine-Dalgarno region to base-pairing by a small regulatory RNA, thus impeding access of the ribosome. (3) Indirect control of mRNA stability. CsrA binds to a target site that upon refolding, impedes access of a ribonuclease. (4) Indirect control of transcriptional termination. CsrA binds to a target site in the mRNA and induces a conformational change that exposes Rho-utilization sequences that can now be exploited by Rho protein to stop transcription elongation of the mRNA. (5) In the absence of CsrA (either due to a mutation that abolishes its function or due to sequestration by CsrA-antagonists, as depicted in Figure 4), a ribosome can recognize the Shine-Dalgarno sequence and initiate mRNA translation. Usually, translationally active mRNAs are protected from the action of ribonucleases.

On the basis of the molecular preference of CsrA binding, and the probability of mRNAs to harbor such sequence/structural recognition motif for CsrA, it is expected that a plethora of mRNAs would be targeted by this protein. This has been recently corroborated by RNA sequencing of the transcripts captured *in vivo* upon crosslinking and affinity purification of CsrA (Holmqvist et al., 2016; Potts et al., 2019). Thus, the proteins of the CsrA family are global regulators of gene expression. Depending on the region of the mRNA where CsrA dimers bind to, the consequence of the interaction may be: (a) translational repression of the mRNA by outcompeting the small ribosomal unit; (b) translational activation upon structural rearrangement of the 5′-untranslated region and exposition of the ribosome binding site; (c) translational regulation by refolding the 5′-UTR such that an sRNA can gain access by base-pairing and prevent ribosomal entry; (d) modulation of mRNA decay by controlling the access of ribonucleases to target sites; (e) modulation of transcription termination (Figure 3) (Romeo and Babitzke, 2019).

## Patchy Distribution of *csrA* Genes Across Eubacteria

**Figure 4:**
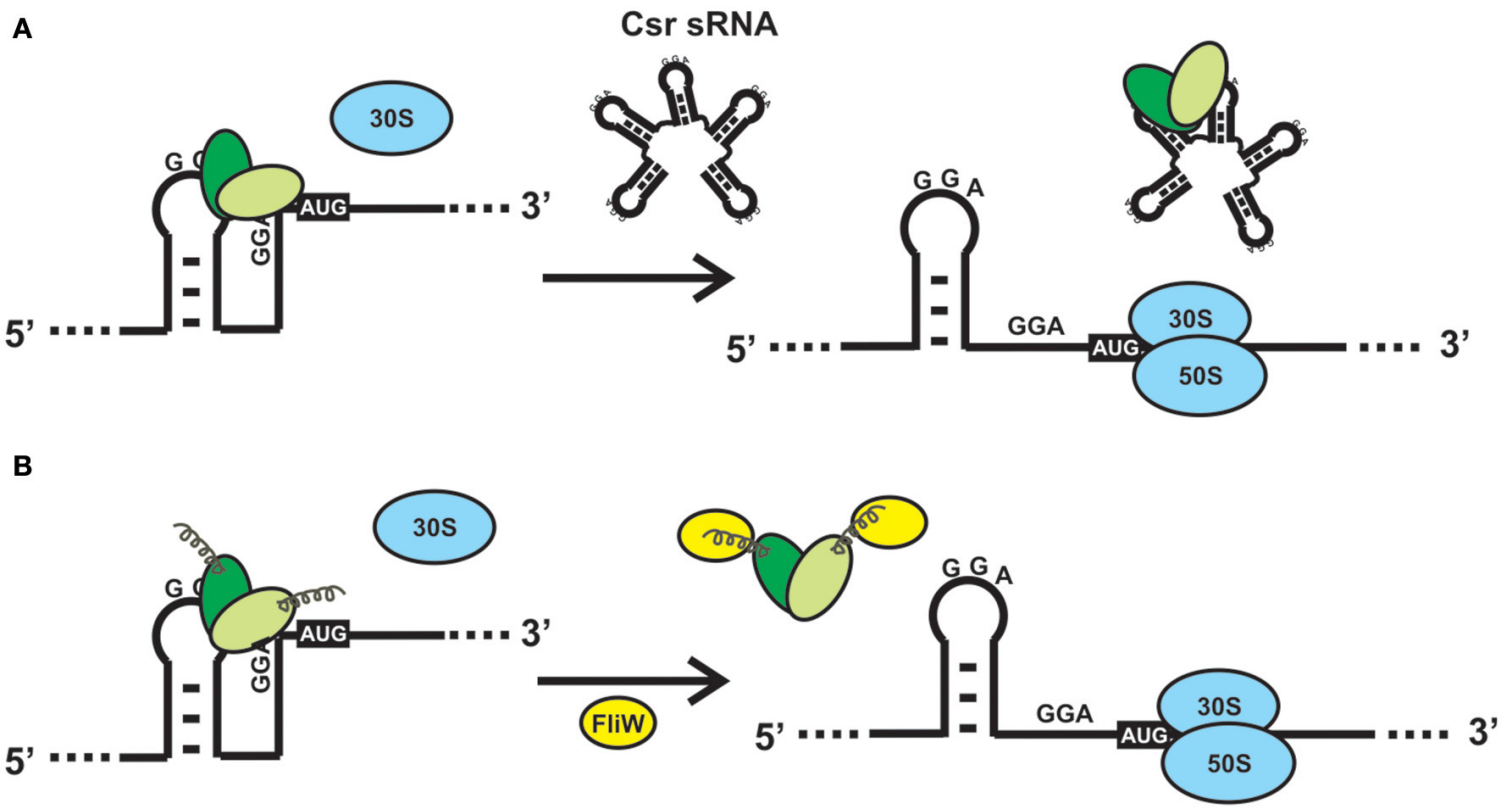
Alternative mechanisms to relieve the direct or indirect effects of CsrA binding to a mRNA. **(A)** Sequestration of CsrA by molecular mimic sRNAs. The sRNAs of the Csr/Rsm family act like protein sponges by offering multiple sequence/structural motifs that fulfill the features shown in Figure 2F. The availability of CsrA dimers for binding to multiple mRNAs is modulated by the intracellular concentration of one or more of these molecular mimic sRNAs. **(B)** CsrA can be displaced from its mRNA substrates upon interaction of a protein like FliW of *B. subtilis* with the extended C-terminal region (Altegoer et al., 2016). Such interaction provokes a conformational change in the protein dimer that reduces the affinity for the RNA.

CsrA-like proteins exhibit a remarkably non-uniform distribution in the eubacterial kingdom (Figure 1). This heterogeneous distribution contrasts with the widespread (although non ubiquitous) presence of other bacterial proteins involved in riboregulatory processes, like Hfq or the endoribonuclease E (RNAse E) (Supplementary Table 1; Sobrero and Valverde, 2012). As for Hfq, CsrA proteins seem to be absent in bacterial species that have adopted an intracellular lifestyle (e.g., *Rickettsia, Chlamydia* and the γ-proteobacterium *Francisella*). Even within the proteobacterial branch, some classes lack CsrA homologs, raising the question about the essentially of this regulatory protein. As we can imagine for every protein-coding gene, its evolutionary trajectory is directly related to its biological function. CsrA structure can serve as a scaffold for protein-RNA or protein-protein interactions, both impacting on gene expression. For instance, in certain lineages, CsrA co-occurs with the protein FliW, a regulator of flagellar gene expression (Mukherjee et al., 2011) (Supplementary Table 1 and Supplementary Figure 1). This co-occurrence can explain the evolution of CsrA in *Bacillus* and ε-proteobacteria (Altegoer et al., 2016), and other bacterial genera presenting both genetic elements. Moreover, the co-occurrence of CsrA and FliW is associated with longer CsrA polypeptides with extended C-termini acting as a binding surface for protein-protein interaction with FliW (Figure 4B).

An intriguing observation is the presence of CsrA homologs in a very limited subset of lineages within the β-proteobacterial clade (Figure 1). Our genomic survey detected only two β-proteobacterial species bearing CsrA homologs: *Bordetella petrii* and *Burkholderia pseudomallei* TSV202 (Supplementary Table 1). *Bordetella petrii* is the only environmental *Bordetella* species hitherto found among the otherwise host-restricted and pathogenic members of the genus *Bordetella* (Von Wintzingerode et al., 2001), and it comprises a group of opportunistic pathogens, mostly associated to lung infections (Mattoo and Cherry, 2005). Interestingly, both *B. petrii* and *Pseudomonas aeruginosa* can share a common niche during the infection of patients suffering of cystic fibrosis (Le Coustumier et al., 2011). In *B. petrii*, we found an annotation (Bpet1351) of 62 amino acids sharing 67% identity with the CsrA counterpart of *P. aeruginosa* strain PAO1 (RsmA). The genomic context of Bpet1351 did not reveal evidences of mobile genetic elements. Thus, Bpet1351 most likely represents a core genetic element of the *B. petrii* genome. As for other β-proteobacteria, we did not detect a homolog of FliW in *B. petrii*. Thus, Bpet1351 is a chromosomally encoded CsrA homolog with a potential role of interaction with RNAs in *B. petrii*. The second β-proteobacterial *csrA* allele is X994_313 in chromosome 1 of *Burkholderia pseudomallei* strain TSV202, which encodes a 79 amino acid polypeptide with a predicted secondary structure consisting in a β_1_β_2_β_3_β_4_α_1_α_2_ topology. Such a slightly longer than the average chain of this protein suggests a possible evolutionary link to the CsrA representatives of ε-proteobacteria or *Bacillus* (Supplementary Table 1); however, the protein encoded by X994_313 is 46% identical to *P. aeruginosa* RsmA (including a clear conservation of the residues involved in protein-RNA interaction) but it is only 25% identical to the CsrA homolog of the ε-proteobacterium *Helicobacter pylori*. Clearly, only an experimental functional approach can give support to the type of regulatory interactions that this CsrA homolog performs in *B. pseudomallei*. Interestingly, a simple inspection of the genomic context of X994_313 reveals the presence of adjacent annotations related to mobile genetic elements.

## How Do Bacteria Relieve the Regulatory Effects of CsrA?

CsrA is a highly abundant molecule in the *E. coli* cytosol, being included within the top 17% of abundant proteins constituted by a group of 179 molecules with more than 2050 copies per cell (Ishihama et al., 2008). The abundance of CsrA is not static, oscillating within 10.000 to 30.000 dimers per cell during the batch growth of *E. coli* (Gudapaty et al., 2001). Similarly, the cellular level of the CsrA ortholog of *Pseudomonas aeruginosa* strain PAO1, RsmA, increases 3-fold in stationary phase (Pessi et al., 2001), whereas the level of the main CsrA ortholog of *P. protegens* strain CHA0 (RsmA) is rather stable along the growth curve (Reimmann et al., 2005). This scenario suggests that there would be a considerable potential for posttranscriptional control by CsrA within the cell, and that it may even increase during growth. Thus, there should be antagonizing mechanisms to relieve the control exerted by CsrA (or its orthologs), when required. Up to date, two mutually exclusive mechanisms for antagonizing the activity of CsrA proteins have been described: 1) molecular mimicry by sRNAs (Figure 4A); 2) allosteric interaction with proteins, like FliW (Figure 4B). The latter has been described in species for which sRNA antagonists have not been found yet, like *Bacillus subtilis* and *Campylobacter jejuni* (Altegoer et al., 2016; Dugar et al., 2016). During the early stages of flagellum assembly in *B. subtilis*, FliW forms a complex with the filament protein Hag; when Hag is secreted to complete the flagellum, FliW is dissociated and it becomes available for interaction with CsrA. At this stage, FliW binds to the C-terminal extension of CsrA and induces the release of the mRNA bound to the RNA pockets, in a non-competitive way (Mukherjee et al., 2016) (Figure 4B). Similar molecular interactions occur between CsrA and CesT in enteropathogenic *E. coli* (Ye et al., 2018). Interestingly, the FliW-CsrA regulatory switch is not only devoted to flagellar gene regulation, because in the absence of FliW, *C. jejuni* experiences differential accumulation of non-flagellar proteins in a CsrA-dependent mechanism (Li et al., 2018).

However, the most pervasive antagonizing strategy to relieve posttranscriptional control by proteins of the CsrA family, is through the expression of molecular mimic sRNAs (Romeo and Babitzke, 2019). This class of non-protein coding regulatory RNA molecules behave as protein sponges that form ribonucleoprotein complexes that temporarily relieve the regulatory effect that CsrA proteins have on their mRNA targets (Figure 4B). To achieve this, molecular mimic sRNAs offer multiple sequence-structural motifs formed by short hairpins exposing unpaired ANGGA pentanucleotides, that is, the preferred molecular target of CsrA proteins (Figures 2F,G). At physiological conditions in which the cellular level of molecular mimic sRNAs is low, proteins of the CsrA superfamily are bound to equivalent motifs typically present in hundreds of target mRNAs. Upon an increase in the intracellular level of the molecular mimic sRNAs, the CsrA-hostage mRNAs are released, and the regulatory effects are reversed (Figure 4A). In essence, the biological function of this class of sRNAs is to modulate the distribution of CsrA dimers into alternative ribonucleoprotein complexes, i.e., CsrA-trapped mRNAs or sRNA-sequestered CsrA dimers (Figure 4A).

In Enterobacteriaceae, the molecular mimic sRNAs that titrate CsrA dimers are referred to as Csr RNAs because, together with the CsrA protein, they were originally characterized as carbon storage regulators (Liu et al., 1997; Weilbacher et al., 2003), whereas in *Pseudomonas* species the so-called Rsm sRNAs were first discovered in association with the CsrA-like counterparts known as RsmA and its close paralogues (Heeb et al., 2002; Valverde et al., 2003; Kay et al., 2005). Csr and Rsm sRNAs are functional and structural homologs that are interchangeable between these γ-proteobacterial taxa (Valverde et al., 2004) but display an enormous divergence at the sequence level with sizes ranging 100 to 400 nt. In addition, from a single gene copy to up to seven functional Csr/Rsm homologs have been detected in different bacterial species (Supplementary Figure 1) (Moll et al., 2010; Lopez-Pliego et al., 2018). Recently, two novel sRNAs with similar sequestering capabilities, RsmV (Janssen et al., 2018) and RsmW (Miller et al., 2016), were discovered in *Pseudomonas aeruginosa*, although in contrast to RsmX, RsmY and RsmZ, their transcription is independent of the GacS-GacA two-component system, and they seem to represent *P. aeruginosa*-specific representatives and are probably of independent evolutionary origin.

## CsrA Proteins and their Molecular Mimic sRNA Partners are Subsidiaries of Signal Transduction Systems

**Figure 5:**
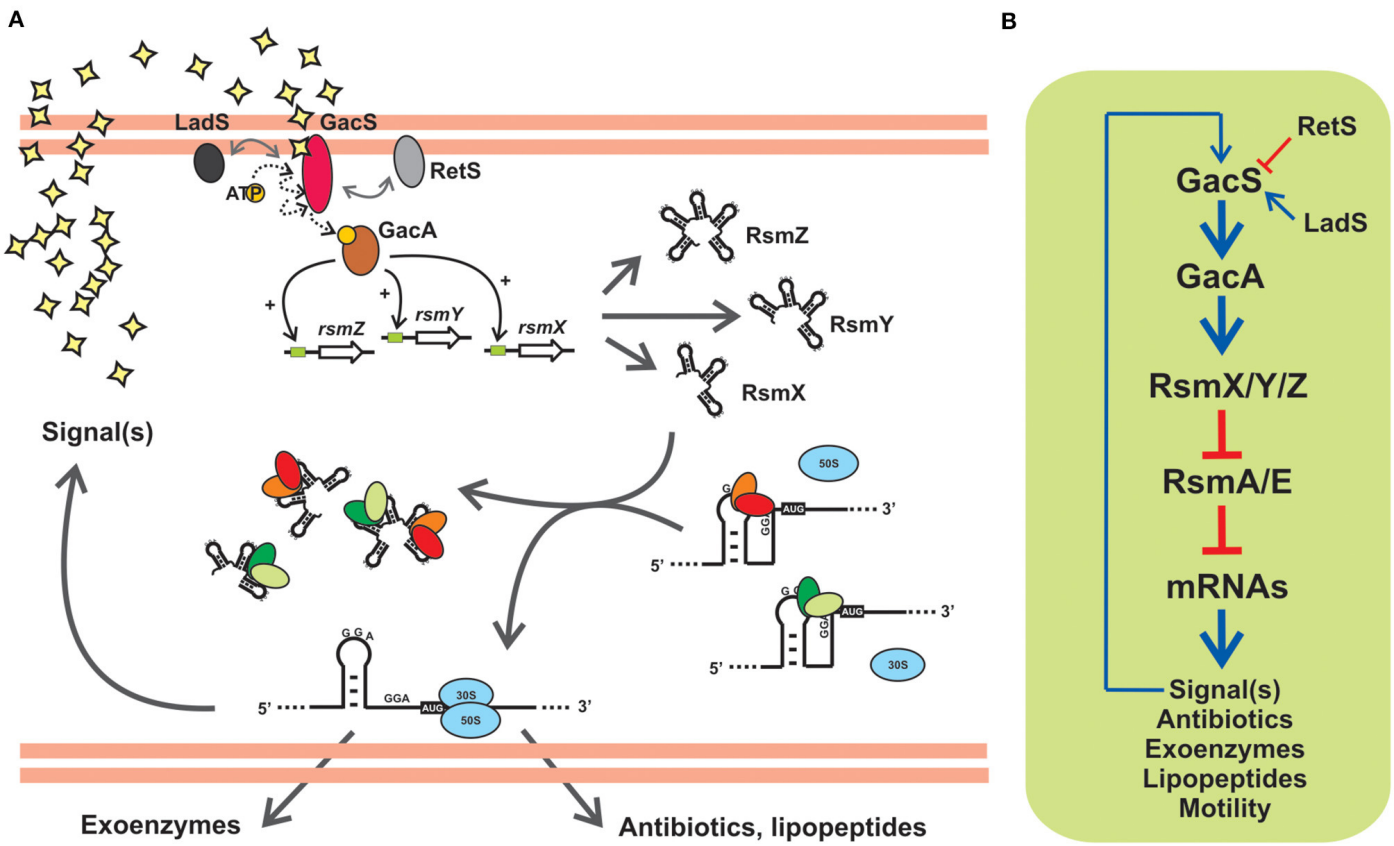
The posttranscriptional Gac-Rsm cascade of *Pseudomonas protegens* strain CHA0: An archetypal signal transduction pathway of γ-proteobacteria controlling the activity of CsrA proteins with molecular mimic sRNAs. **(A)** In strain CHA0, the GacS-GacA TCS responds to uncharacterized autoinducing signals, and promotes translation of various mRNAs involved in exoproduct formation and control of plant root pathogens (antibiotics, lipopeptides, extracellular lytic enzymes), by relieving the translational blockage caused by the two CsrA homologs RsmA and RsmE. This is achieved by activating transcription of *rsmX, rsmY* and *rsmZ* genes, thereby increasing the intracellular abundance of the three sponge RNAs RsmX, RsmY and RsmZ, that sequester RsmA and RsmE. Two accessory orphan sensor kinases, RetS and LadS, modulate the activity of GacS. **(B)** Schematic linear representation of the Gac-Rsm regulatory pathway of *P. protegens* strain CHA0. The biosynthesis of a diffusible signal compound is also under the control of the cascade, as a number of other phenotypes contributing to the antifungal ability of this strain, and it represents a positive feedback input for the pathway. This diagram, as well as the scheme in **(A)**, is simplified and omits modulatory aspects like the requirement of RsmA/E proteins for a proper expression of *rsmX/Y/Z* genes (Valverde et al., 2003; Kay et al., 2005). The number of components in the posttranscriptional module formed by Rsm proteins and Rsm sRNAs, as well as the nature of the target mRNAs, vary between different species (see Tables 1, 2).

With the high cellular level of CsrA dimers fluctuating within a relatively narrow range, the role of antagonizing molecules becomes critical to relieve posttranscriptional control of target mRNAs. To achieve this, different bacterial taxa have recruited two-component sensory-transducing systems (TCS) to manipulate the intracellular level of the molecular mimic sRNAs of the Csr type (Table 1) (Valverde and Haas, 2008). In these circuits, mainly characterized for members of the γ- proteobacteria, an environmental stimulus is perceived by the membrane-bound sensor protein that modulates the phosphorylation status of the partner transcriptional factor; the latter then controls the expression of the gene(s) encoding the molecular mimic sRNA(s), which in turn will adjust the cellular level of these sRNAs to modulate the balance of CsrA distribution between target mRNAs and sRNAs (Figure 4A). The nature of the stimulus and the complexity of the signal transduction cascade (i.e., the number of CsrA protein and Csr sRNA homologs) vary between different species (Tables 1, 2; Supplementary Figure 1), but the basic architecture of the regulatory cascade is similar: a TCS converts a physicochemical input into a primary transcriptional output (i.e., activation of sRNA gene expression), then it is converted into a subsequent global posttranscriptional reversion of the pre-existing effect of CsrA dimers over multiple mRNAs (Figure 5).

**Table 1:** Signal transducing two-component systems and their cognate posttranscriptional modules involving CsrA homologs and CsrA-antagonistic sRNAs in γ-proteobacteria.

**Species**	**TCS**	**Stimulus**	CsrA homolog^*^	Molecular mimic sRNA(s)^*^	**Controlled phenotypes**	**References**
*Acinetobacter baumannii*	GacS-GacA	Unkown	[CsrA]	[RsmX, RsmY, RsmZ]	Pili, motility, biofilm, metabolism of aromatic compounds, virulence	Kulkarni et al., 2006; Cerqueira et al., 2014
*Azotobacter vinelandii*	GacS-GacA	Unknown	RsmA	RsmZ1-7, RsmY1-2	Alginate, PHB and alkyl resorcinol lipid biosynthesis	Lopez-Pliego et al., 2018, 2020
*Erwinia amylovora*	GrrS-GrrA	Unknown	RsmA	RsmB	Flagella, T3SS, amylovoran biosynthesis	Ancona et al., 2016
*Escherichia coli*	BarA-UvrY	Acetate, formate	CsrA	CsrB, CsrC	Glycogen synthesis, gluconeogenesis, motility, biofilm	Chavez et al., 2010
*Halomonas anticariensis*	GacS-GacA	Unknown	[CsrA]	Unknown	Quorum sensing, exopolysaccharide, biofilm.	Tahrioui et al., 2013
*Legionella pneumophila*	LetS-LetA	Unknown	CsrA	RsmY, RsmZ	>40 effector proteins secreted by T4SS (cytotoxicity), motility	Nevo et al., 2014; Feldheim et al., 2018
*Pectobacterium carotovorum*	ExpS-ExpA	Unknown	RsmA	RsmB	Extracellular lytic enzymes, T3SS-secreted harpin	Cui et al., 2001
*Salmonella enterica* serovar Typhimurium	BarA-SirA	Acetate, formate	CsrA	CsrB, CsrC	Stress resistance, virulence (HilD), aerobic and nitrate respiration, motility, fimbriae	Lawhon et al., 2002; Zere et al., 2015
*Serratia mascescems*	GacS-GacA	Unknown	CsrA	CsrB, CsrC	Motility, biofilm, coral mucus utilization	Krediet et al., 2013; Ito et al., 2014
*Vibrio cholerae*	VarS-VarA	Unknown	CsrA	CsrB, CsrC, CsrD	Virulence, biofilm, quorum sensing	Lenz et al., 2005; Butz et al., 2019
*Vibrio fischeri*	GacS-GacA	Citrate?	CsrA	CsrB, CsrC, CsrD	Luminiscence, siderophores, motility	Septer et al., 2015
*Vibrio tasmaniensis*	VarS-VarA	Unknown	CsrA	CsrB1-4	Metalloproteases	Nguyen et al., 2018

* *Proteins or sRNAs between brackets were found by genome inspection and have not been experimentally characterized yet*.

**Table 2:** Diversity of the posttranscriptional Csr(Rsm) regulatory module of the Gac-Rsm cascade in the genus *Pseudomonas*.

***Pseudomonas* species—strain**	**Additional factors controlling Gac-Rsm cascade**	**CsrA homolog(s)**	**Molecular mimic sRNA(s)**	**Controlled phenotypes**	**References**
*P. aeruginosa* PAO1	LadS, RetS and PA1611 histidine kinases	RsmA, RsmN (RsmF)	RsmY, RsmZ, RsmW (Gac-independent), RsmV (Gac-independent)	Quorum sensing; virulence factors, sessile-to-biofilm switch	Ventre et al., 2006; Marden et al., 2013; Morris et al., 2013; Chambonnier et al., 2016; Miller et al., 2016; Schulmeyer et al., 2016; Janssen et al., 2018; Romero et al., 2018
*P. brassicacearum* NFM421	Unknown	RsmA, RsmE	RsmX, RsmY, RsmZ	Antibiotics, indole acetate, extracellular enzymes, quorum sensing, T6SS, alginate, biofilm	Lalaouna et al., 2012
*P. chlororaphis* 30-84	Unknown	RsmA, RsmE	RsmX, RsmY, RsmZ	Biosynthesis of phenazines, quorum sensing, extracellular enzymes	Chancey et al., 1999; Wang et al., 2013
*P. donghuensis* HYS/SVBP6/P482	Unknown	RsmA, RsmE, Rsm3	RsmY, RsmZ	Biosynthesis of 7-hydroxytropolone, antifungal activity, production of HCN and other volatile compounds	Yu et al., 2014; Ossowicki et al., 2017; Agaras et al., 2018; Chen et al., 2018; Muzio et al., 2020
*P. entomophila* Pe	Unknown	RsmA1, RsmA2, RsmA3	RsmY, RsmZ	Insect virulence factors, exopotease, lipopeptide	Vodovar et al., 2006; Vallet-Gely et al., 2010
*P. fluorescens* SS101	Unknown	RsmA, RsmE	RsmY, RsmZ	Lipopeptide, iron acquisition, motility, chemotaxis T6SS	Song et al., 2015
*P. protegens* CHA0	Temperature, ppGpp, Krebs cycle intermediates, LadS, RetS	RsmA, RsmE	RsmX, RsmY, RsmZ	Biocontrol properties (antifungal compounds, HCN, extracellular enzymes, lipopeptide)	Heeb et al., 2002; Valverde et al., 2003; Reimmann et al., 2005; Humair et al., 2009; Takeuchi et al., 2009; Workentine et al., 2009; Sobrero et al., 2017
*P. putida* KT2440	Unknown	RsmA, RsmE, RsmI	RsmY, RsmZ, RsmX?	Motility, biofilm formation (LapA adhesin)	Martinez-Gil et al., 2014; Huertas-Rosales et al., 2016
*P. syringae* pv *syringae* DC3000	LadS, RetS	CsrA1 (RsmI), CsrA2 (RsmA), CsrA3 (RsmE), CsrA4, CsrA5	RsmX1-5, RsmY, RsmZ	Carbon metabolism, virulence, motility, production of secondary metabolism, quorum sensing	Moll et al., 2010; Records and Gross, 2010; Ferreiro et al., 2018

The cascades come in different flavors. For instance, in *E. coli*, the TCS BarA-UvrY responds to acetate and formate, as well as to the medium pH (Mondragon et al., 2006; Chavez et al., 2010), to activate the expression of *csrB* and *csrC* encoding the two molecular mimic sRNAs CsrB and CsrC, bearing 18 and 11 CsrA binding sites, respectively (Liu et al., 1997; Weilbacher et al., 2003), which in turn modulate the availability of the single CsrA protein of this species; the outcome of the cascade activation is the regulation of carbon polymer biosynthesis, motility and biofilm formation (Romeo and Babitzke, 2019). In the plant pathogen *Pectobacterium wasabiae* (formerly *Erwinia carotovora*), the BarA-UvrY homolog TCS named ExpS-ExpA controls expression of a single molecular mimic sRNA RsmB (containing 19 recognition motifs) to interact with a single CsrA ortholog (RsmA); the major characterized regulatory targets are a series of extracellular plant cell wall-degrading enzymes (Mukherjee et al., 1996). In *Vibrio cholerae*, the TCS VarS-VarA regulates the activity of a single CsrA protein through the activation of three genes encoding the molecular mimic sRNAs CsrB, CsrC, and CsrD (with 23 to 28 putative recognition motifs); the cascade has a major impact on the expression of the quorum sensing system, thus indirectly controlling biofilm formation and virulence (Lenz et al., 2005). So far, proteins of the two-component system formed by BarA/GacS/VarS and UvrY/GacA/VarA families are a hallmark of γ-proteobacterial taxa (Supplementary Table 1).

## The Gac-Rsm Cascade of *Pseudomonas* Species

The genus *Pseudomonas* comprises over 250 defined species, with a remarkable distribution across major ecological niches, either as free-living or in close interactions with animals, insects, plants and fungi, at relatively mild or extreme conditions (Palleroni, 2015). An important number of species are opportunistic pathogens of clinical relevance for humans, and of economic concern in animal and plant production; however, there are also an important number of species with probiotic traits for animals and plants, and representatives with metabolic capacities for bioremediation purposes (Palleroni, 2015). Their ease to isolate and culture in the lab has resulted in the availability of an important wealth of physiological, biochemical, genetic, and genomic resources. All the accesible *Pseudomonas* genomes reveal the existence of a posttranscriptional regulatory cascade having the general features described in the previous section, which is known as the Gac-Rsm system.

Gac stands for global antibiotic and cyanide control (Laville et al., 1992) and Rsm stands form regulator of secondary metabolism (Blumer et al., 1999). The two regulatory phenomena were discovered in the plant-probiotic isolate *P. protegens* (ex-*fluorescens*) CHA0 and linked to each other upon the identification of the gene *rsmA* encoding the CsrA ortholog RsmA (Blumer et al., 1999). The prototypic backbone of the Gac-Rsm cascade of *Pseudomonas* is illustrated in Figure 5A for the case of *P. protegens* CHA0. The sensor kinase GacS responds to a solvent-extractable extracellular compound (of yet unknown chemistry) produced by the cells and that accumulates as a function of cell density in batch cultures, and most likely autophosphorylates and then transfers a phosphoryl moiety to the GacA transcriptional regulator. Phosphorylated GacA, or an intermediary factor, act on DNA sequences upstream the promoters of the *rsmX, rsmY* and *rsmZ* genes to activate their transcription, which results in the intracellular accumulation of the corresponding molecular mimic sRNAs. At the end of the pathway, the two RNA-binding proteins of the CsrA family, RsmA and RsmE, are titrated away from their complexes with different target mRNAs to form inactive dead-end ribonucleoprotein assemblages with the sponge sRNAs. As a consequence of this cascade of molecular events, the mRNAs encoding proteins and enzymes required for the synthesis of a variety of secondary metabolites are engaged into translation (Figure 5A). At the population level, the activation of the Gac-Rsm cascade in *P. protegens* CHA0 provokes a coordinated behavioral change which is evidenced as a marked increase (and social sharing) in the biosynthesis of antifungal compounds, extracellular enzymes, cyclic lipopeptides (Lapouge et al., 2008; Sobrero et al., 2017), and interestingly, of the GacS-inducing signal itself (Dubuis et al., 2006), which implies a positive-forward loop (Figure 5), As deduced from the linearity of the cascade (Figure 5B) and the molecular mechanism of each of the components, deleting the *rsmA* and *rsmE* genes provokes activation of all these biosynthetic pathways ahead of time, an observation that originated the naming of the CsrA homologs of *P. protegens* CHA0 as RsmA first, and RsmE some years later (Heeb et al., 2002; Reimmann et al., 2005). Additionally, deleting either *gacS* or *gacA*, impede the removal of RsmA and RsmE from their target mRNAs, and such single mutations logically result in a global fall in the production of all secondary metabolites, a phenotype that inspired naming of the components of the signal-transducing cascade GacS-GacA (Laville et al., 1992; Zuber et al., 2003). At the ecological level, the cascade is key for the cells to avoid predation by eukaryotic bacterivores that dislike some of the extracellular compounds (Jousset et al., 2006), and, at the same time, it confers protection from fungal pathogens to the roots of plants that are colonized by this strain (Lapouge et al., 2008). Hence, the Gac-Rsm cascade of *P. protegens* CHA0 has deep implications at the individual, collective and ecological levels.

The backbone of the Gac-Rsm cascade -as detailed for strain CHA0- is conserved in the genus *Pseudomonas*, but it presents several species-specific variations: first, the number of components downstream of the TCS GacS-GacA is highly variable in terms of the number of molecular mimic sRNA molecules and of CsrA homologs (the latter will be thoroughly discussed in the next sections) (Table 2); second, there are additional factors that modulate the functioning of the cascade (Table 2 and Supplementary Table 1); third, the regulon and/or the main characterized phenotypes that are subject to Gac-Rsm control differ across species and determine the outcome of their interactions with other bacteria or with eukaryotic hosts (Table 2).

With regard to the occurrence of additional factors influencing the Gac-Rsm cascade, two additional sensor kinases have been reported to modulate the activity of GacS directly: LadS, that stimulates GacS activity, and RetS, that is a negative regulator (Figure 5, Table 2) (Ventre et al., 2006; Chambonnier et al., 2016). Whereas, the master components of the cascade GacS and GacA are broadly distributed within the γ-proteobacteria, LadS and RetS show a more sporadic occurrence (Supplementary Table 1). Both genes encoding LadS and RetS are present together with *gacS* and *gacA* homologs only in *Pseudomonas, Lysobacter*, and *Alcanivorax* species (Supplementary Table 1). GacS and GacA are present together with LadS (but not with RetS) in five γ-proteobacterial genera (*Alteromonas, Halomonas, Idiomarina, Marinibacter*, and *Pseudoalteromonas*); conversely, GacS, GacA, and RetS (but not LadS) co-occur only in *Azotobacter* species (Supplementary Table 1). Thus, although not exclusively restricted to members of the genus *Pseudomonas*, the genes encoding the accessory sensor histidine kinases LadS and RetS are present together with *gacS* and *gacA* in a few γ-proteobacterial taxa, suggesting that they have emerged recently in the evolutionary history of these closely related genera.

When inspecting the occurrence of homolog genetic elements that constitute the Gac-Rsm cascade in the KEGG database (Supplementary Table 1), we found the surprising and unexpected case of the Gram-positive bacterium *Streptococcus dysgalactiae* subsp. *equisimilis* having ortholog genes encoding the BarA/GacS/VarS sensor kinase (locus tag NCTC11565_05684; 62% identical to *P. protegens* GacS), the UvrY/GacA/VarA response regulator (locus tag NCTC11565_03616; 87% identical to *P. protegens* GacA), and two CsrA proteins (locus tags NCTC11565_05400 and NCTC11565_00091; 81% and 30% identical to *P. protegens* GacA, respectively) (Supplementary Table 1). Worth mentioning is the fact that the most similar *csrA* allele to that of *P. protegens rsmA*, displays the same local genetic arrangement as in *Pseudomonas* genomes (*alaS*-*lysA*-*csrA*-*tRNA*^Ser^-*tRNA*^Arg^), and it is immediately flanked downstream by a large cluster of genes of a Mu-like prophage. No other Gram-positive species contains a set of genes so closely resembling that of the Gac-Rsm cascade of γ-proteobacteria (Supplementary Table 1).

## How Many Different CsrA (Rsm) Orthologs in *Pseudomonas*?

Up to date, dozens of articles have reported on the impact of CsrA proteins in bacterial fitness by reverse genetics approaches (Table 1). The isolation of *csrA* deletion mutants in many different bacterial species demonstrates that *csrA* is not an essential gene. The essentially of *csrA* has been only argued in *E. coli*, which develops a conditional growth behavior in the presence of a glycolitic carbon source (Timmermans and Van Melderen, 2009). Interestingly, whereas the ample majority of the genomes we have inspected have only one CsrA homolog, there are few lineages in which at least two independent *csrA*-like copies are found. This is the special case of the genera *Pseudomonas* and *Legionella*. The latter holds the highest average number of CsrA paralogues per genome (Supplementary Table 1) (Chien et al., 2004; Abbott et al., 2015). From now on, we will focus in the multiplicity of CsrA paralogues in the *Pseudomonas* pangenome.

In contrast to the rest of γ-proteobacterial branches other than *Legionella*, more than 90% of *Pseudomonas* species present at least two *csrA* alleles per genome (Supplementary Tables 1, 3). Historically, the first experimentally characterized representatives in the genus *Pseudomonas* were designated *rsmA* in *P. aeruginosa* strain PAO1 (Blumer et al., 1999) and in *P. protegens* (ex-*fluorescens*) strain CHA0 (Heeb et al., 2002). However, the *Pseudomonas* RsmA proteins are structural and functional homologs of the enterobacterial CsrA protein (see above; Figure 2). For historical reasons, in the next paragraphs we will stick to the Rsm naming when discussing the phylogenetic aspects of these proteins in the genus *Pseudomonas*.

**Figure 6:**
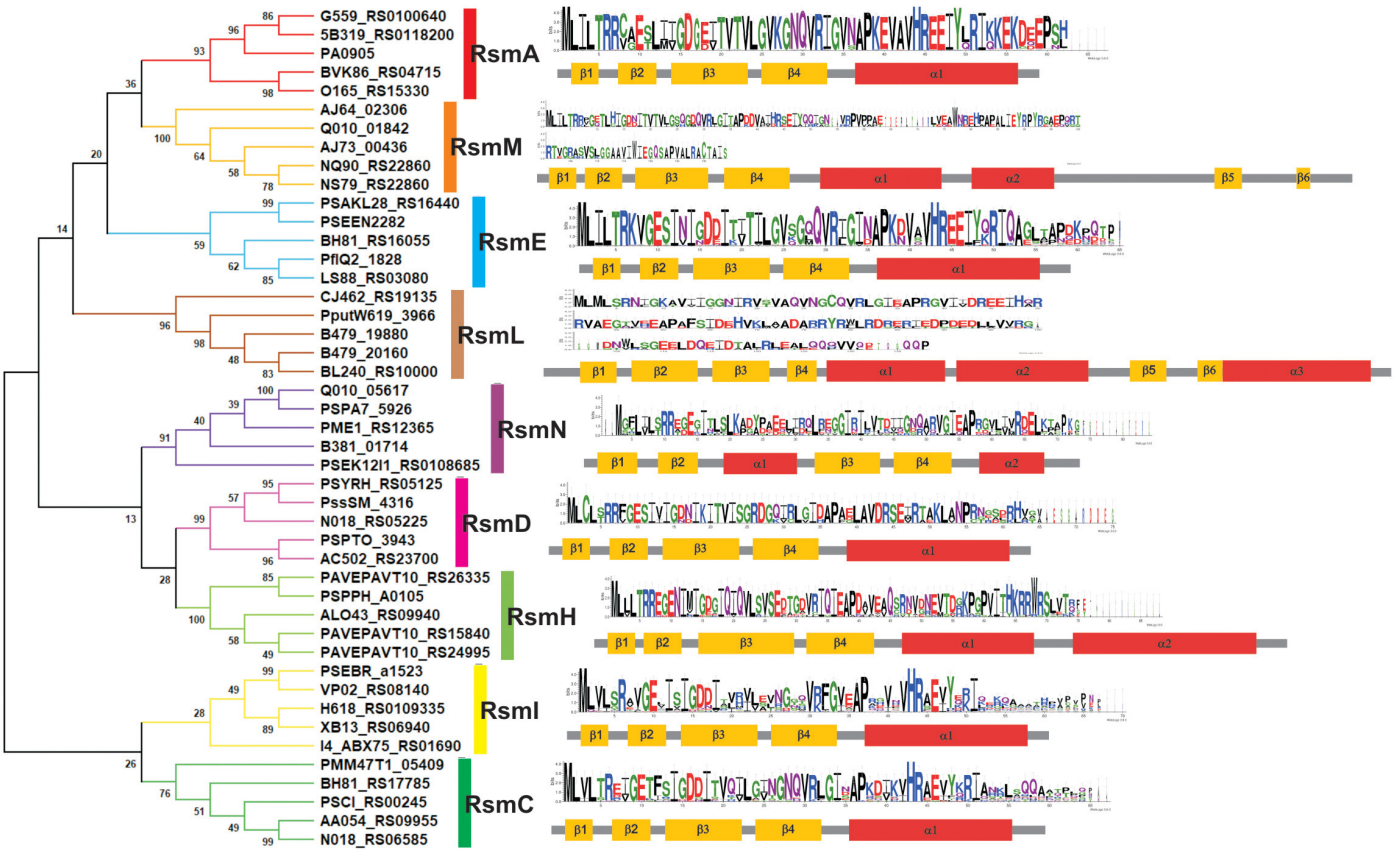
Families of Rsm (CsrA) proteins within the genus *Pseudomonas* as deduced from comparative genomics. Phylogenetic relationships of the 9 subfamilies of *Pseudomonas* Rsm proteins. The evolutionary history was inferred using the Neighbor-Joining method (Saitou and Nei, 1987). The optimal tree with the sum of branch length = 7.34 is shown. The percentage of replicate trees in which the associated taxa clustered together in the bootstrap test (1000 replicates) are shown next to the branches (Felsenstein, 1985). The evolutionary distances were computed using the JTT matrix-based method (Jones et al., 1992) and are in the units of the number of amino acid substitutions per site. This analysis involved 45 amino acid sequences. For each one, the locus tag identification is presented in the figure. All positions containing gaps and missing data were eliminated (complete deletion option). There was a total of 46 positions in the final dataset. Evolutionary analyses were conducted in MEGA X (Kumar et al., 2018). Secondary structure was predicted with JPRED4 server (Drozdetskiy et al., 2015). For each Rsm subfamily, a sequence logo (Crooks et al., 2004) was generated from multiple alignment data by MUSCLE (Edgar, 2004).

The developers of the *Pseudomonas* Genome Database (www.pseudomonas.com) have generated *Pseudomonas*-specific orthologous groups (POGs) based on protein sequence-similarity algorithms (Buchfink et al., 2015). This sensitive and fast tool allowed us to fetch a detailed list of protein homologs from available genomes using a query sequence. As a starting point, we used the amino acid sequence of RsmA from the *P. aeruginosa* PAO1 annotation PA0905, and we retrieved 7 different POGs (Supplementary Table 2). In order to obtain a more precise picture of the evolution of these groups, we randomly extracted 10 sequences from each POG and inferred their phylogenetic relationship by Neighbor Joining (NJ) (Supplementary Figure 2). A simplified version of this analysis, with only 5 sequences per POG, is presented in Figure 6. The NJ inference actually shows the existence of 9 different Rsm protein subfamilies within the genus *Pseudomonas*. If we consider that each POG represents a standalone protein subfamily, our phylogenetic analysis has uncovered two novel subfamilies not previously detected in the *Pseudomonas* Genome Database. Four out of the 9 groups correspond to the well characterized paralogue subfamilies RsmA (Blumer et al., 1999; Pessi et al., 2001), RsmE (Reimmann et al., 2005), RsmI (Huertas-Rosales et al., 2016) and RsmN (also referred to as RsmF in the literature) (Marden et al., 2013; Morris et al., 2013). On the basis of the POG definition in the *Pseudomonas* database and of our NJ clustering, we propose the novel paralogue subfamilies RsmC, RsmD, RsmH, RsmL and RsmM (Figure 6). Interestingly, all the paralogue groups present a high degree of conservation of the amino acids involved in the interaction with RNA molecules (at least for nucleotide contacts reported for RsmE Schubert et al., 2007) (Supplementary Figure 3). Except for RsmN (Marden et al., 2013; Morris et al., 2013), the rest of *Pseudomonas* Rsm subfamilies present the archetypical topologic fold β_1_β_2_β_3_β_4_α_1_ (Figure 2B), with some minor variations (Figure 6). The first noticeable difference among the subfamilies is the polypeptide size, which occurs particularly at the C-terminal region. As expected, this extension at the C-terminus has an impact on protein topology (Figure 6). RsmM and RsmH members present an additional alpha helix, whilst RsmL members have 2 extra helices; RsmM and RsmL also show two additional short β-sheets (Figure 6). The fact that these extended C-terminal regions does not seem to be unstructured in these subfamilies, suggests that it may have an important contribution to protein stability and/or in protein networking. By analogy to the CsrA homologs that interact with FliW (Figure 4, Supplementary Table 1), the extended C-terminal regions of RsmM, RsmH, and RsmL could function as a scaffold region for protein-protein interactions. In fact, there are no experimental evidences yet of which are the direct or indirect proteins interactors of members of the CsrA superfamily. Because of its major role in posttranscriptional control of mRNAs, most efforts have been directed to reveal the RNA interactome of CsrA proteins, but it may be expected that accessory proteins would be recruited to the CsrA-RNA ribonucleoprotein complex, like Hfq and the degradosome (Sorger-Domenigg et al., 2007; De Lay et al., 2013; Bruce et al., 2018).

## Phylogenetic Distribution of the Rsm Subfamilies Within the Genus *Pseudomonas*

**Figure 7:**
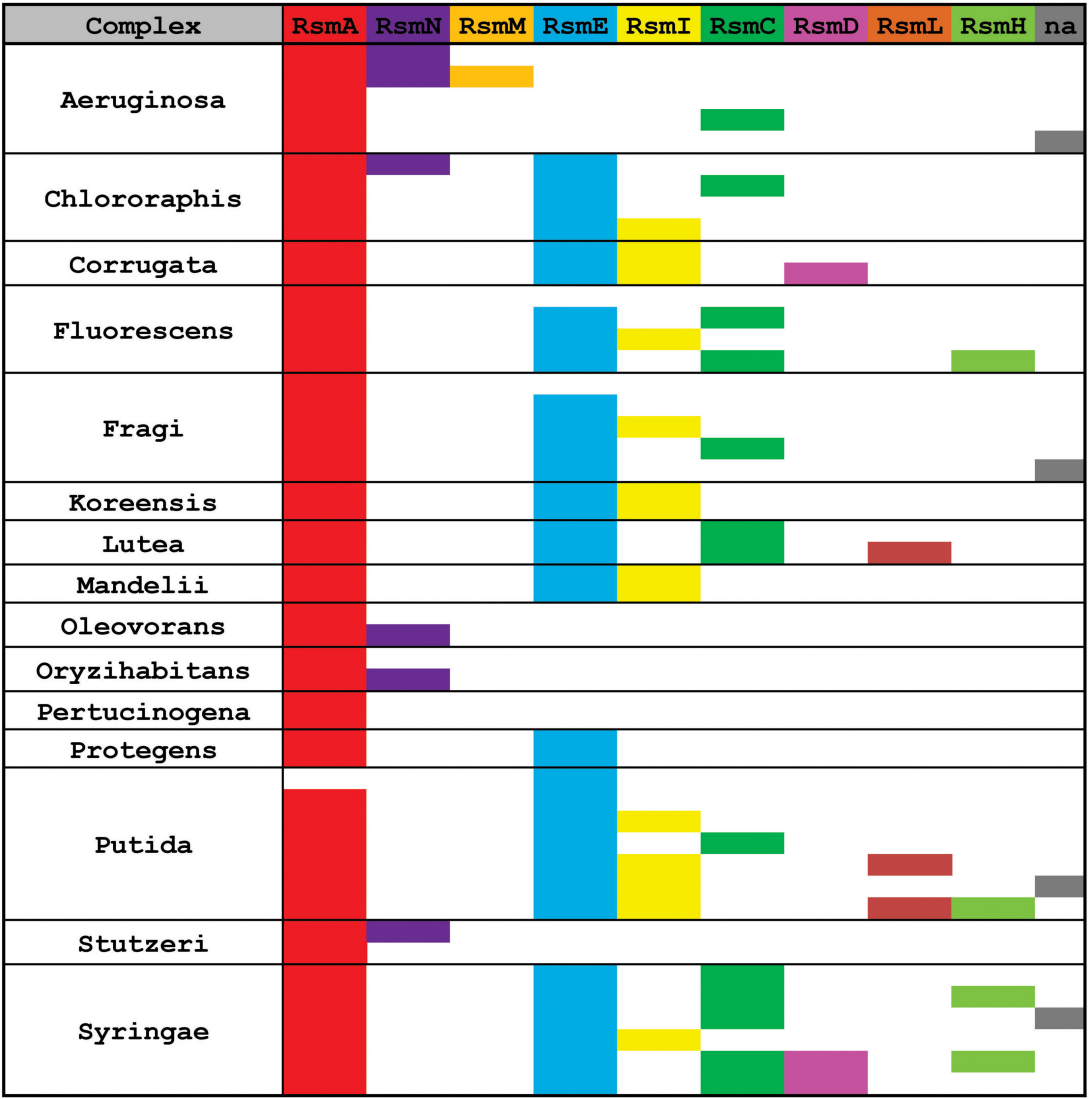
Distribution of Rsm paralogues in the major *Pseudomonas* taxonomic subgroups. Within each subgroup, each row represents different species or strains displaying different combinations of alleles (see extended data in Supplementary Table 4). n.a., the sequence of the *rsm* paralogue could not be assigned with enough confidence to any of the 9 subfamilies.

Once we defined the number of Rsm paralogue subfamilies of the genus (Figure 6), we set out to explore the distribution of each paralogue group among the different *Pseudomonas* species. The genetic diversity and metabolic flexibility of the genus has contributed to the successful colonization of a broad variety of ecosystems in our planet (Silby et al., 2011). Besides, an important number of species interact with most eukaryotic taxa (Silby et al., 2011), contributing to their health or their disease (Mercado-Blanco and Bakker, 2007; Loper et al., 2012; Winstanley et al., 2016). Thus, the distribution of Rsm paralogues among different species, each with different niches and lifestyles, might be related to its function. We then inspected 137 *Pseudomonas* genomes (Supplementary Table 3), trying to cover all the reported taxonomic groups (Peix et al., 2018). We applied BLASTP under non-restrictive conditions with *P. aeruginosa* RsmA as the bait, in order to retrieve the different paralogues from each genome (Supplementary Tables 3, 4). Next, we added each retrieved paralogue sequence to the dataset used for our NJ phylogenetic analysis so that we could infer which Rsm subfamily it belongs to (Supplementary Table 5). We here present the distribution of Rsm paralogues for 8 different taxonomic clades, which roughly represent the diversity of the genus (Figure 7; for an extended version, see Supplementary Figure 4).

RsmA is the most broadly distributed Rsm subfamily (Figure 7). Almost all *Pseudomonas* genomes have one *rsmA* allele. RsmE appears as the second most represented subfamily. Yet, *P. aeruginosa* and its close relatives (like *P. stutzeri* and *P. oryzihabitants*) do not have RsmE paralogues (Figure 7). Instead, it appears that RsmN has become the RsmA sidekick within the *P. aeruginosa, P. stutzeri, P. oleovorans* and *P. oryzihabitants* branches (Figure 7). Much to our surprise, we found an *rsmN* homolog in the chromosome of a strain phylogenetically distant from the former 4 species, *P. chlororaphis* HT66 (Supplementary Table 4). However, the contig that contains the *rsmN* allele (locus tag M217_RS0107790) does not reveal clear evidences of association to mobile genetic elements that would explain an interspecies lateral transfer. In contrast, the *rsmN* homolog (locus tag PME1_RS12365) of *P. mendocina* NBRC 14162 (belonging to the Oleovorans clade) is found nearby an integrase gene (PME1_RS12365) located just downstream a tRNA gene (PME1_RS12285). This genetic organization is typical of genomic islands (Williams, 2002). As we did not find this genetic arrangement in neither of the other *rsmN* homologs, the case of *P. mendocina* NBRC 14162 is unusual in that *rsmN* lies within a possible genomic island.

Another striking observation of the distribution of Rsm subfamilies, is the fact that there is no single genome containing both RsmI and RsmC; rather, we found either RsmI or RsmC within a genome (Figure 7). As both homologs share a common ancestor (Figure 6), this is a clear example of a putative gene duplication and speciation event, for which the host or environmental constraints have shaped the pathway and consolidated either one of the two homologs. Nevertheless, RsmI and RsmC have a fairly broad distribution across the genus (Figure 7).

In contrast to the rather ample distribution of RsmA, RsmE, RsmC, and RsmI, the rest of the *Pseudomonas*' Rsm subfamilies have a much more restricted distribution (Figure 7). RsmL is present only in some members of the *P. putida* and *P. lutea* branches, RsmD in some species within the *P. syringae* and *P. corrugata* clades, and RsmH only in a few species within the *P. syringae, P. putida*, and *P. fluorescens* groups (Figure 7 and Supplementary Table 5). Notably, a subset of *P. aeruginosa* genomes bears a third Rsm paralogue, RsmM, in addition to RsmA and RsmN (Figure 7). As members of the RsmM paralogue subfamily were not detected out of *P. aeruginosa*, RsmM is a species-specific Rsm paralogue.

Finally, we have detected the presence of multiple copies of alleles from the same subfamily; for instance, two *rsmA* alleles in *P. corrugata* and three *rsmH* genes in *P. tolaasii* (Supplementary Table 4). In summary, the distribution pattern of Rsm paralogues in the chromosomes of the genus *Pseudomonas* is another trait that reflects the dynamic plasticity of their genomes and provides evidences of evolutionary genetic processes at different stages of progress.

## Heavy Traffic of CsrA (Rsm) Homologs in *Pseudomonas* Plasmids and Phages

To the best of our knowledge, only two scientific reports have revealed the presence of *csrA* alleles in plasmids from environmental bacterial isolates. The first one described a *csrA* allele in the replication region of the cryptic plasmid pMBA19a from a *Sinorhizobium meliloti* environmental isolate (Agaras et al., 2013). The encoded CsrA homolog has a high degree of identity with typical γ-proteobacterial CsrA proteins but presents a C-terminal extension that may fold into an extra α-helix (Agaras et al., 2013). Genetic complementation of Δ*rsm* mutants from *P, aeruginosa* PA01 or *P. protegens* CHA0 suggests that the plasmidic *S. meliloti* homolog is functional and that the extra α-helix is most likely involved in protein stability and that it does not interfere with mRNA binding and riboregulatory functions (Agaras et al., 2013). The second case is a *csrA*-like sequence (PSLF89_RS34715) also found in a cryptic plasmid from the fish pathogen *Piscirickettsia salmonis* (Ortiz-Severin et al., 2019). *P. salmonis* is a γ-proteobacterium related to the intracellularly adapted species of *Coxiella, Francisella* and *Wolbachia* genera (Fryer et al., 1992). The authors suggested that the plasmidic *csrA* allele could be involved in the regulation of the expression of other plasmidic genes related to a conjugative-type secretion system, by analogy to the genetic context of a *csrA* allele of two genomic islands of *Legionella pneumophila* Corby (Glockner et al., 2008). Nevertheless, the actual role of these *csrA* elements for the fitness of each plasmid remains obscure.

In contrast to plasmids, there is no single report on the presence of *csrA* alleles in phage genomes. A hint pointing to the possible association of *csrA* genes with phages comes from the inspection of the genomic context of the *csrA* allele X994_313 of *Burkholderia pseudomallei* TSV202, which shows several genes encoding functions related to genetic mobility like type IV secretion system (X994_330), conjugative transfer (X994_308), and a phage-related protein (X994_344). An exhaustive functional analysis of the X994_313 genomic context would be required to reveal if this genetic cluster represent a new class of putative mobile genetic element, such as phage-inducible chromosomal islands (Penades and Christie, 2015).

Although few in number, the findings discussed above suggest a possible contribution of mobile genetic elements and horizontal gene transfer (HGT) mechanisms to the dynamic evolution of CsrA-dependent regulatory pathways. Moreover, the presence of *csrA*-like alleles could be itself linked to the transfer process, as proposed for the CsrA homologs of in *Legionella pneumophila*, denoted as CsrT, which are co-inherited with type IV secretion system genes in all known integrative and conjugative elements (ICEs) of legionellae; it was suggested that CsrT regulate ICE activity to increase their horizontal spread (Abbott et al., 2015). Nevertheless, the sole horizontal movement of a *csrA* allele and its incorporation into the genetic wealth of a receptor bacterium does not guarantee its establishment, and furthermore, to be maintained it requires to be recruited by pre-existing regulatory networks and confer a fitness benefit for the new host cell. Examples of how sRNAs, and their RNA auxiliary proteins like Hfq or RNAse E, contribute to riboregulatory processes in the interplay of core genomic elements and foreign DNA acquired by HGT can be found elsewhere (Frohlich and Papenfort, 2016). Thus, at this point, we asked ourselves whether the presence of *csrA* alleles in plasmids and phages is a rare phenomenon, or if instead, it is a quantifiable property of *crsA* genes.

**Figure 8:**
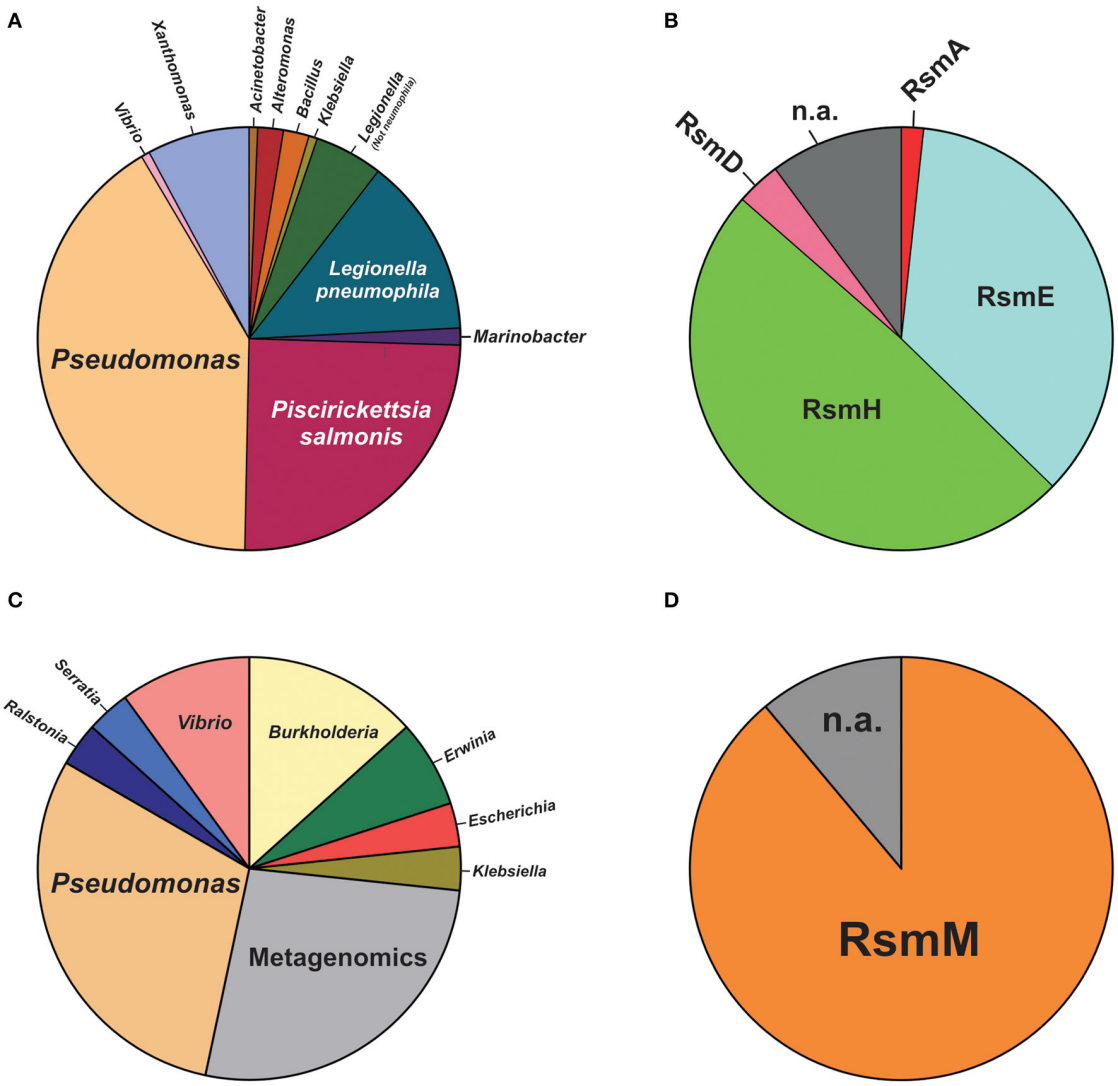
Species distribution of CsrA homologs in natural plasmids and phages of the genus *Pseudomonas*. **(A)** Pie chart illustrating the proportional distribution (%) of plasmids deposited in the PLSDB database (https://ccb-microbe.cs.uni-saarland.de/plsdb/) bearing a *csrA* allele and hosted by different eubacterial host taxa. The total number of plasmids is 161 (for details, see Supplementary Table 6). **(B)** Distribution of Rsm paralogues within the 61 plasmids hosted by *Pseudomonas* species. n.a.: not assigned. **(C)** Distribution (%) of phages deposited in the NCBI viral genomes database (https://www.ncbi.nlm.nih.gov/genomes/GenomesGroup.cgi?taxid=10239) bearing a *csrA* allele and hosted by different eubacterial host taxa. The total number of phages is 30 (for details, see Supplementary Table 7). **(D)** Distribution of Rsm paralogues within the 9 phage genomes hosted by *Pseudomonas* species. n.a., not assigned.

For plasmids, we carried out a tBLASTn search in the plasmid database PLSDB (Galata et al., 2019), using the *E. coli csrA* gene sequence as query (minimum query coverage of 30%). Among 20668 natural plasmids in the database, we found 161 (0.8%) containing a *csrA* allele (Supplementary Table 6). The phylogenetic distribution of *csrA*^+^ plasmids is shown in Figure 8. The genera with the highest number of *csrA*^+^ plasmids were *Pseudomonas* (63; 39%), *Piscirikketsia* (38; 24%), *Legionella* (29; 18%) and *Xanthomonas* (12; 8%) (Figure 8A). If we consider the total number of plasmids deposited in the database for each genus, it comes out that the lineages with the highest proportion of *csrA*^+^ are *Legionella* (29 *csrA*^+^ out of 92 plasmids; 32%) and *Pseudomonas* (63 *csrA*^+^ out of 330 plasmids; 19%). Within *Pseudomonas*, by far, the species with the highest number of *csrA*^+^ plasmids are *P. syringae* (18/63) and *P. aeruginosa* (15/63); the rest of the *csrA*^+^ plasmids of the genus are distributed in another 17 species (Figure 8B, Supplementary Table 6). Notably, the most frequent paralogues present in *Pseudomonas* natural plasmids were RsmH (49%) and RsmE (36%) (Figure 8B), being the RsmE paralogues exclusively associated with plasmids hosted by representatives of the *P. aeruginosa* cluster, whereas the RsmH paralogues were exclusively present in plasmids hosted by representatives of the *P. syringae* cluster (Supplementary Table 6). Two interesting observations derive from these findings. First, the paralogues present in plasmids of the *P. aeruginosa* lineage (RsmE-type) have not been detected yet in the *P. aeruginosa* chromosomes, for which the *rsmE* type of paralogues seem to have been evolutionary excluded (Figure 7). Second, the paralogues present in plasmids of the *P. syringae* lineage (RsmH-type) have been found in only a fraction of the *P. syringae* chromosomes (Figure 7), suggesting that (in contrast to *rsmA* and *rsmE* alleles) *rsmH* is not part of the core genome of *P. syringae*. In support of the latter hypothesis, a micro-genomic context analysis of different *rsmH* alleles let us reveal the presence of genes encoding relaxases and mobilization proteins. One example is *P. caripapayae* ICMP2855, in which the *rsmH* homolog (ALO80_RS00560) is located nearby genes encoding a resolvase (ALO80_RS00585), a relaxase (ALO80_RS00615) and a plasmid mobilization protein (ALO80_RS00610). Another illustrative example is that of the environmental isolate *P. brassicacearum* LZ-4, which belongs to the Corrugata group (Huang et al., 2017) and that represents, according our genome mining analysis, the unique example of a *Pseudomonas* isolate bearing an *rsmH* homolog outside the Syringae clade (Supplementary Table 5). The genomic context of this *rsmH* homolog (LZ_RS24290) includes genes for a putative relaxase (LZ_RS24345) and a conjugal transfer protein (LZ_RS24350). Altogether, these observations bring us closer to a plausible link between RsmH paralogues and conjugative mobile elements, due to the neighboring association with genes encoding resolvases and integrases. In summary, it appears that there is an important proportion of *Pseudomonas* natural plasmids that contain genes encoding members of the CsrA (Rsm) family of posttranscriptional regulatory proteins, with RsmH and RsmE being the most frequent types (Figure 8).

For phages, we interrogated the NCBI non-redundant protein database with PSI-BLAST using the *E. coli* CsrA sequence as query and narrowing the search to the Viruses taxid:10239. After 10 iterations, the list of hits saturated in 30 phages among a total of 2517 bacteriophage genomes (1.2%). The list of detected *csrA*^+^ phages is shown in Table 3. Interestingly, the genus with the highest number of *csrA*^+^ phages is *Pseudomonas* (9) (Figure 8C) and most of them (8) correspond to *P. aeruginosa* isolates (Table 3). The proportion of *Pseudomonas* phages deposited in the NCBI database that contain a *csrA* allele is 9 out of 185 (5%). Remarkably, all 8 *P. aeruginosa* phages contain an allele of the *rsmM* type (Figure 8B, Supplementary Table 7), an Rsm subfamily that we only found in the chromosomes of a subset of *P. aeruginosa* isolates (Figure 7), like strain Hex1T that contains an *rsmM* allele (ABI36_RS00125) in a genomic locus flanked by genes reminiscent of phages (ABI36_RS00135 and downstream genes). We hypothesize that a similar scenario would be possible for members of the RsmD and RsmL sub-families; in both cases, we found alleles in genomic contexts flanked by phage-related genes, consistent with prophages or phage remnants. Thus, the evidences presented here for the first time, strongly suggest that members of the genus *Pseudomonas* are active in shuttling *csrA* alleles in mobile genetic elements, including (pro)phages, conjugative elements and plasmids.

**Table 3:** Csr homologs in bacterial phages.

**Phage**	**Host bacteria**	**Score**	**Query cover**	**E value**	**Identity**	**Genbank accession**	**References**
AUS531phi	*Pseudomonas aeruginosa*	80.1	90%	1,00E-19	63%	QGF21359.1	
YMC11/07/P54_PAE_BP	*Pseudomonas aeruginosa*	80.1	90%	1,00E-19	63%	YP_009273743.1	
vB_Pae_BR153a]	*Pseudomonas aeruginosa*	79.7	90%	2,00E-19	62%	QBI80752.1	Tariq et al., 2019
phi297	*Pseudomonas aeruginosa*	79.0	90%	3,00E-19	62%	YP_005098048.1	Krylov et al., 2013
vB_Pae_CF24a	*Pseudomonas aeruginosa*	74.0	95%	3,00E-17	57%	QBI82405.1	Tariq et al., 2019
H66	*Pseudomonas aeruginosa*	73.6	95%	4,00E-17	55%	YP_009638943.1	
F116	*Pseudomonas aeruginosa*	72.8	95%	8,00E-17	55%	YP_164288.1	Byrne and Kropinski, 2005
vB_Pae_CF63a	*Pseudomonas aeruginosa*	72.4	95%	1,00E-16	55%	QBI82448.1	Tariq et al., 2019
1.232.O._10N.261.51.E11	*Vibrio tasmaniensis*	68.2	93%	1,00E-15	59%	AUR96800.1	
Prokaryotic dsDNA virus sp.	Marine metagenome	67.8	98%	2,00E-15	53%	QDP60892.1	Roux et al., 2016
Caudovirales phage	Male skin metagenome	64.3	88%	5,00E-14	50%	ASN71505.1	
Prokaryotic dsDNA virus sp.	Marine metagenome	63.2	93%	1,00E-13	42%	QDP52503.1	
Marine virus	Marine metagenome	63.6	96%	9,00E-13	30%	AKH48332.1	Chow et al., 2015
GP4	*Ralstonia solanacearum*	61.2	96%	9,00E-13	25%	AXG67699.1	Wang et al., 2019
DC1	*Burkholderia cepacia* LMG 18821	60.9	98%	1,00E-12	28%	YP_006589976.1	Lynch et al., 2012
VvAW1	*Vibrio vulnificus*	60.5	98%	2,00E-12	52%	YP_007518368.1	Nigro et al., 2012
Bcep22	*Burkholderia cenocepacia*	58.9	96%	6,00E-12	30%	NP_944283.1	Gill et al., 2011
Parlo	*Serratia* sp.	57.4	98%	3,00E-11	31%	QBQ72211.1	Bockoven et al., 2019
Bcepil02	*Burkholderia cenocepacia*	57.0	96%	4,00E-11	32%	YP_002922721.1	Gill et al., 2011
Bcepmigl	*Burkholderia cenocepacia*	56.6	96%	5,00E-11	32%	YP_007236796.1	
PEp14	*Erwinia pyrifoliae*	56.6	95%	6,00E-11	27%	YP_005098413.1	
SopranoGao_25	*Klebsiella pneumoniae* 51503	56.2	93%	9,00E-11	26%	ASV45048.1	Gao et al., 2017
Prokaryotic dsDNA virus sp.	Marine metagenome	55.1	96%	2,00E-10	40%	QDP56147.1	Roux et al., 2016
Pavtok	*Erwinia amylovora*	54.7	90%	3,00E-10	29%	AXF51437.1	
Skulduggery	*Pseudomonas fluorescens* SBW25	53.9	85%	6,00E-10	32%	ARV77111.1	Wojtus et al., 2017
Prokaryotic dsDNA virus sp.	Marine metagenome	53.5	90%	9,00E-10	28%	QDP46120.1	Roux et al., 2016
VH2_2019	*Vibrio natriegens*	55.1	81%	1,00E-09	41%	QHJ74590.1	
vB_EcoP_PTXU04	*Escherichia coli*	53.2	72%	1,00E-09	36%	QBQ76642.1	Korf et al., 2019
uvMED	Mediterranean Sea metagenome	47.4	83%	2,00E-07	29%	BAR35878.1	Mizuno et al., 2013
Prokaryotic dsDNA virus sp.	Marine metagenome	41.6	72%	3,00E-05	36%	QDP60323.1	Roux et al., 2016

*CsrA homologs were identified with the NCBI PSI-BLAST tool using the E. coli CsrA sequence as query and narrowing the database search to the Viruses taxid:10239 (database accessed on April 2, 2020). A total of 10 iterations was needed until no further hits were added to the results list*.

Another interesting feature of the search of *csrA* alleles in phage genomes, is the fact that we detected bacterial lineages acting as hosts for *csrA*^+^ phages that do not contain chromosomal *csrA* genes; this is the case of *Burkholderia cepacia* and *Burkholderia cenocepacia* (Table 3). This situation is similar to that of the *csrA* gene detected in the plasmid pMBA19a hosted in a bacterial species (*S. meliloti*) devoid of chromosomal *csrA* homologs (Agaras et al., 2013).

Circumstantial associations between CsrA proteins and mobile genetic elements other than plasmids and phages, have also been reported. In addition to the case of CsrT and the ICEs of *L. pneumophila* (Abbott et al., 2015), several integrative elements of the Gram-negative anaerobic pathogen *Dichelobacter nodosus* have insertion target sites adjacent to the chromosomal *csrA* gene (Cheetham et al., 2008), whereas in *Serratia* sp. ATCC 39006, CsrA controls prophage activation (Wilf et al., 2013). These findings, and the significant presence of *csrA* alleles in plasmids and phages reported here (Figure 8, Table 3, Supplementary Tables 6, 7), altogether strongly suggest that members of the CsrA family are important regulatory factors for the success of mobile genetic elements, and that there is a fairly substantial pool of *csrA* genes in the bacterial mobilome.

## Concluding Remarks

Members of the CsrA family are small dimeric RNA binding proteins apparently restricted to Eubacterial species, but with a non-uniform distribution (Figure 1). The preferred target RNA motif is a relatively short hairpin with an unpaired GGA triplet in the loop. Each CsrA dimer can bind simultaneously to two of these target RNA motifs (Figure 2). Hundreds of cellular mRNAs contain one or more target motifs, which expose them to CsrA binding and its subsequent regulatory effects. Depending on the position of the RNA motif, CsrA may control translation efficiency, transcript stability of transcription termination (Figure 3). The regulation by CsrA may be reversed by antagonizing proteins (like FliW or CesT), or more often by molecular mimic sRNAs that offer multiple target sites so that CsrA dimers become sequestered in large ribonucleoprotein complexes (Figure 4). The cellular concentration of the antagonistic sRNAs is typically modulated by signal transducing cascades mastered by TCSs of the BarA/GacS-UvrY/GacA type (Figure 5, Table 1).

Two intriguing aspects of the biology of the CsrA family are its variegated presence in different phylogenetic lineages and its disparate multiplicity of alleles, the latter occurring particularly in certain γ-proteobacterial lineages like *Xanthomonas, Legionella*, and *Pseudomonas* (Figure 1). Within the *Pseudomonas* genus, the CsrA protein homologs and their antagonistic sRNAs have been thoroughly characterized for *P. aeruginosa* and *P. protegens*, although exploration of other species revealed an increasing diversity of CsrA (Rsm) proteins (Table 2). Here, by exploiting the available genomic resources of the genus, we identified novel subfamilies of Rsm proteins (Figure 6). We propose that the *Pseudomonas* pangenome contains 9 subfamilies of Rsm proteins, of which 5 are reported here for the first time: RsmC, RsmD, RsmH, RsmL, and RsmM (Figure 6). Despite their sequence variability, they all share conserved residues that are relevant for RNA-binding (Supplementary Figure 3) thus suggesting they are all involved in posttranscriptional regulation of gene expression. When inspecting the distribution of paralogues among species, it appears that RsmA and RsmE are the most widespread representatives, followed by RsmC and RsmI (Figure 7). On the other hand, one paralogue subfamily seems to be lineage-specific -RsmM for *P. aeruginosa*- whereas two others are restricted to a few lineages: RsmL in *P. putida* and *P. lutea*, and RsmH and RsmD mostly in *P. syringae* (Figure 7). Strikingly, the closely related paralogues RsmI and RsmC never coexist in the same lineages, pointing to a possible exclusion phenomenon (Figure 7).

An important feature uncovered in this genomic survey is the conspicuous presence of *csrA* alleles in natural plasmids and bacteriophages (Table 3, Figure 8, Supplementary Tables 6, 7), confirming and extending previous secluded observations (Agaras et al., 2013; Ortiz-Severin et al., 2019). Of interest are our findings that there seems to be a bias toward the presence of certain types of *rsm* alleles in plasmids (encoding RsmH or RsmE) and in phages (RsmM) from *Pseudomonas* strains (Figure 8). Overall, these findings strongly suggest that CsrA proteins are instrumental for the fitness of mobile genetic elements, and as a side effect, to increase the rate of dispersal and speciation of these proteins. Loss-of-function experiments are required to test the requirement of CsrA proteins for mobility and establishment of plasmids and phages.

An overall view of the distribution of Rsm subfamilies in the chromosomes and in the extrachromosomal genetic pool of the genus (Figures 6–8, Table 3, Supplementary Tables 6, 7 and Supplementary Figure 4) suggests that the RsmA subfamily is the ancestor paralogue of the genus *Pseudomonas* that was inherited from a γ-proteobacterial predecessor. As suggested in a previous report (Morris et al., 2013), a duplication and speciation event gave origin to the RsmE and RsmN paralogue subfamilies, being RsmE subsequently spread into most lineages other than *P. aeruginosa*. We propose that within the *P. aeruginosa* lineage, a more recent duplication and speciation event originated the *rsmM* allele that was co-opted by species-specific phages. Most likely, a second wave of duplications and speciation from *rsmA* gave origin to *rsmC, rsmH*, and *rsmI* subfamilies, which have a patchier genomic distribution than RsmA and RsmE. Of these, *rsmE* and *rsmH* succeeded to be incorporated into species-specific plasmids from different lineages. Finally, the more recently evolved chromosomal paralogues would be *rsmD* and *rsmL*, which are restricted to a few lineages and that may have an alien origin from phages. Overall, our *Pseudomonas* dataset shows that this genus possesses a core set of Rsm genes, and a set of accessory Rsm types strongly associated with mobile genetic elements.

The multiplicity of *rsm* alleles per chromosome in *Pseudomonas* species (Figure 7, Supplementary Figure 4) does not have an obvious explanation. It implies, however, that (if expressed) all alleles are functional and contribute to bacterial fitness, and that the layer of posttranscriptional regulation under Rsm control in *Pseudomonas* lineages is key. Despite the significant contribution of gene duplication to bacterial evolution, the increase of the genome size represents a cost for the organism. So, there must be a balancing evolutionary force to retain the duplicate gene. Instead of performing a new function, the paralogue functions under a different condition (Lynch and Force, 2000). In this context, the multiplicity of paralogues in *Pseudomonas* genomes may offer the possibility of defining subsets of target mRNAs that require differential regulation under distinct environmental or cellular conditions. What clearly follows as a perspective to obtain support to this hypothesis is the need to characterize the function of all Rsm paralogues in a genome, their expression pattern, their interaction with sRNA antagonists, and their corresponding set of target mRNAs.

## Author Contributions

PS and CV conceived and designed the article, performed the database and bibliographic searches, and prepared tables and figures. All authors shared writing of the manuscript, revised it and approved the submitted version.

## Conflict of Interest

The authors declare that the research was conducted in the absence of any commercial or financial relationships that could be construed as a potential conflict of interest.

## Supplementary Material

Supplementary Figure 1 Phylogenetic distribution of genes encoding CsrA, FliW, Hfq, RNAse and UvrY proteins in Eubacteria. The heat map shows the presence of homolog genes for each considered protein in representative species from different classes in the Eubacterial kingdom. At the bottom of the Figure, the heat scale denotes the number of paralogues present per genome.

Supplementary Figure 2 Phylogenetic inference defining the families of Rsm paralogues within the genus *Pseudomonas*. The evolutionary history was inferred using the Neighbor-Joining method (Saitou and Nei, 1987). The optimal tree with the sum of branch length = 9.08 is shown. This analysis involved 75 amino acid sequences. For each sequence, the locus tag identification is presented in the figure. All positions containing gaps and missing data were eliminated (complete deletion option). There was a total of 42 positions in the final dataset. Evolutionary analyses were conducted in MEGA X (Kumar et al., 2018).

Supplementary Figure 3 Amino acid conservation across all families of *Pseudomonas* Rsm paralogues. A multiple alignment was generated with MUSCLE (Edgar, 2004) containing all the 45 Rsm paralogues used for the phylogenetical inference (see Figure 6). The sequence LOGO is the graphical representation of this multiple alignment.

Supplementary Figure 4 Phylogenetic distribution of members of the Rsm paralogue subfamilies within the genus *Pseudomonas*. The phylogeny of the genus was built using the sequence of the *gyrB* gene. The evolutionary history was inferred using the Neighbor-Joining method (Saitou and Nei, 1987). The optimal tree with the sum of branch length = 5.65 is shown. The evolutionary distances were computed using the Kimura 2-parameter method (Kimura, 1980) and are in units of number of base substitutions per site. This analysis involved 138 nucleotide sequences. All positions with <95% site coverage were eliminated, i.e., fewer than 5% alignment gaps, missing data, and ambiguous bases were allowed at any position (partial deletion option). There were a total of 2,414 positions in the final dataset. Evolutionary analyses were conducted in MEGA X (Kumar et al., 2018). For each *Pseudomonas* strain, the subfamily of Rsm paralogues present in the corresponding genome is indicated with colored filled circles, following the color scheme shown at the bottom of the figure.

Supplementary Table 1 Phylogenetic distribution of genes encoding CsrA, FliW, Hfq, RNAse, BarA, LadS, RetS and UvrY proteins in Eubacteria. Data was obtained from KEGG database (Kanehisa and Goto, 2000). Briefly, we inspected the presence of each gene using the KEGG ontology tool: *csrA* (K03563), *barA* (K07678), *ladS* (K20971), *retS* (K20972), *uvrY* (K07689), *rne* (K08300), *hfq* (K03366) and *fliW* (K13626).

Supplementary Table 2 Number of proteins of each Rsm subfamily detected in the *Pseudomonas* genome database. The dataset was generated using the *Pseudomonas* orthologous groups (POGs) from the *Pseudomonas* genome database (Winsor et al., 2016). Non annotated Rsm paralogues within the POGs were identified by interrogating all available *Pseudomonas* genomes with BLASTP using the amino acid sequence of RsmA from *Pseudomonas aeruginosa* (PA0905) as query (Expect value cut-off = 1).

Supplementary Table 3 Number of Rsm paralogues per genome in *Pseudomonas* species. The table lists the number of different Rsm paralogues present in the chromosome of each *Pseudomonas* species studied in this work. For some species (e.g., *P. aeruginosa* or *P. putida*), we included several different strains, some of them bearing different combinations of paralogues.

Supplementary Table 4 Amino acid sequence of Rsm paralogues in *Pseudomonas* species. For paralogue identification, we used a phylogenetic inference approach based on the analysis presented in Figure 6. Each sequence was evaluated under the same phylogenetical analysis to assign it into an Rsm subfamily.

Supplementary Table 5 Distribution of Rsm paralogues in the major *Pseudomonas* taxonomic subgroups. The table details the presence of an allele of each different Rsm subfamily in the genome of the *Pseudomonas* species and strains listed in Supplementary Table 4.

Supplementary Table 6 CsrA homologs in natural plasmids. The unpublished *csrA* alleles in natural plasmids were identified with the tBLASTn tool of the plasmid database PLSDB v2020_03_04 (Galata et al., 2019), accessed on April 2, 2020, using the *E. coli csrA* gene sequence as query, and setting a minimum query coverage of 30%.

Supplementary Table 7 CsrA homologs in phages. The unpublished *csrA* alleles in natural phages were identified by querying the NCBI non-redundant protein database with PSI-BLAST using the *E. coli* CsrA sequence as a bait and narrowing the search to the Viruses taxid:10239 (accessed on April 2, 2020). After 10 iterations, the hit list reached a plateau with 30 identified sequences.
