# Supplementary figures and images for: Comparative Genomics and Evolutionary Analysis of RNA-Binding Proteins of the CsrA Family in the Genus Pseudomonas
Source: Front Mol Biosci. 2020 Jul 10;7:127. doi: 10.3389/fmolb.2020.00127 (PMC7366521; doi:10.3389/fmolb.2020.00127)

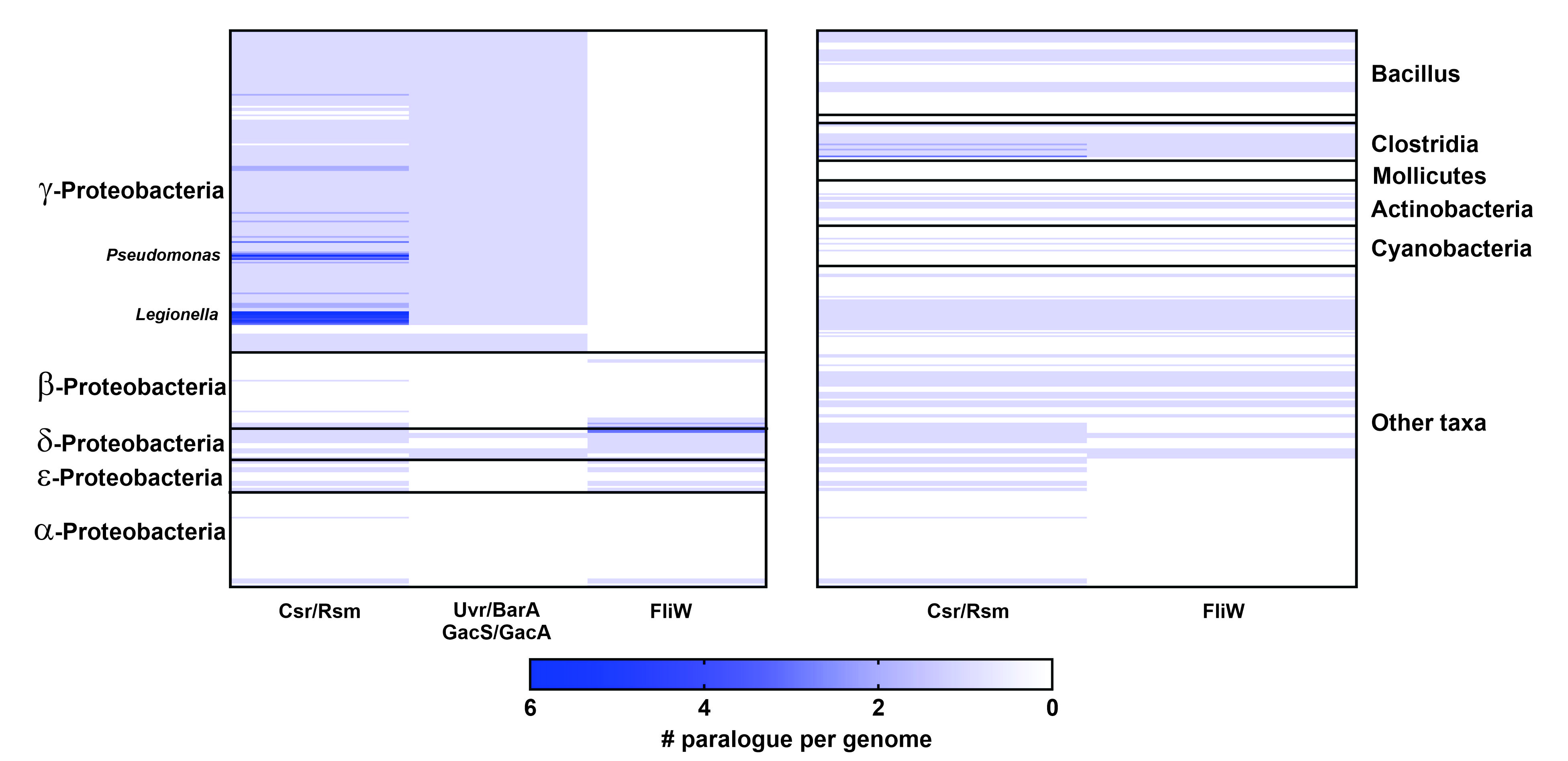

Supplement: Supplementary Figure 1 — Phylogenetic distribution of genes encoding CsrA, FliW, Hfq, RNAse and UvrY proteins in Eubacteria. The heat map shows the presence of homolog genes for each considered protein in representative species from different classes in the Eubacterial kingdom. At the bottom of the Figure, the heat scale denotes the number of paralogues present per genome. [file Image_1.JPEG]

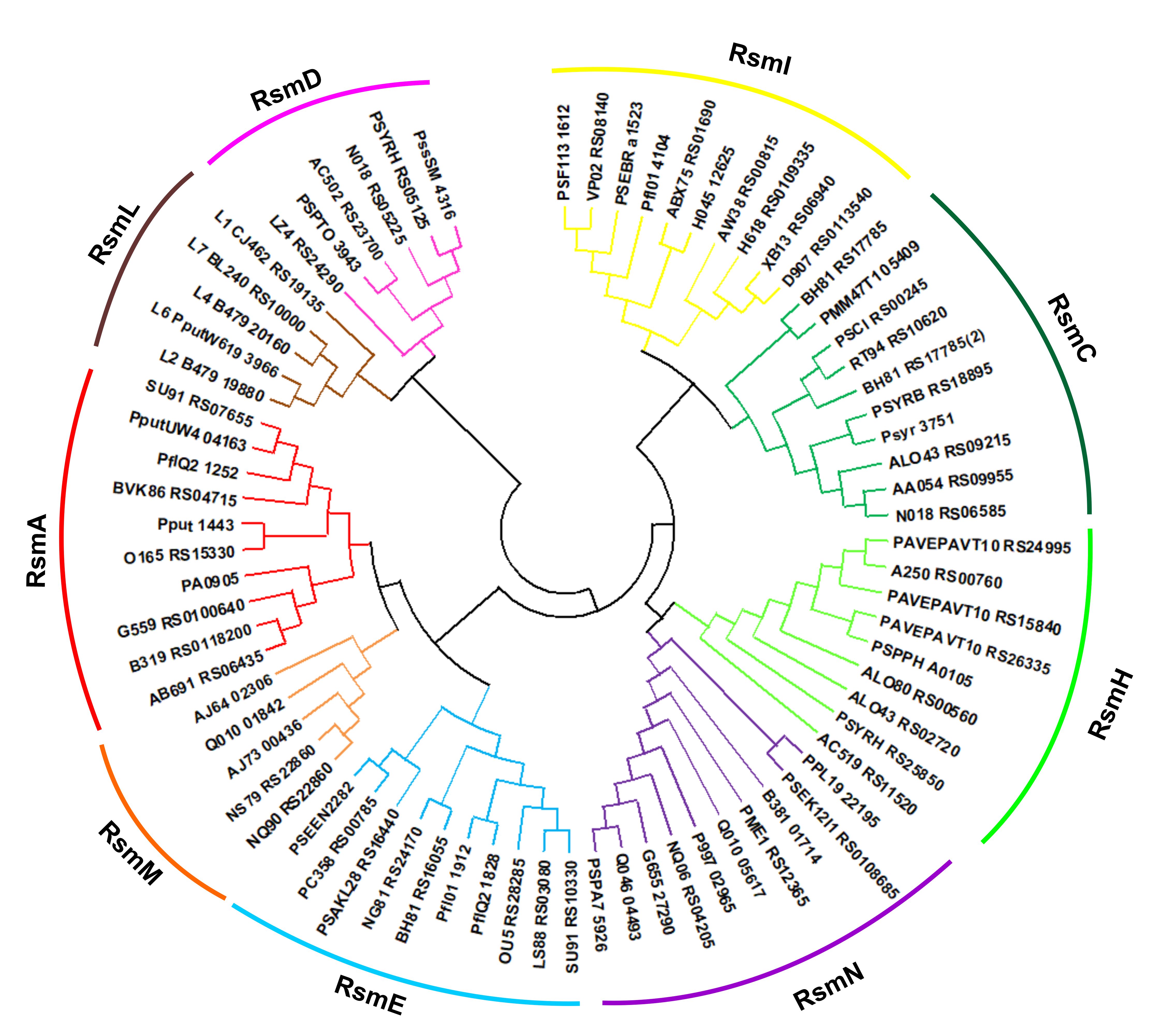

Supplement: Supplementary Figure 2 — Phylogenetic inference defining the families of Rsm paralogues within the genus Pseudomonas. The evolutionary history was inferred using the Neighbor-Joining method (Saitou and Nei, 1987). The optimal tree with the sum of branch length = 9.08 is shown. This analysis involved 75 amino acid sequences. For each sequence, the locus tag identification is presented in the figure. All positions containing gaps and missing data were eliminated (complete deletion option). There was a total of 42 positions in the final dataset. Evolutionary analyses were conducted in MEGA X (Kumar et al., 2018). [file Image_2.JPEG]

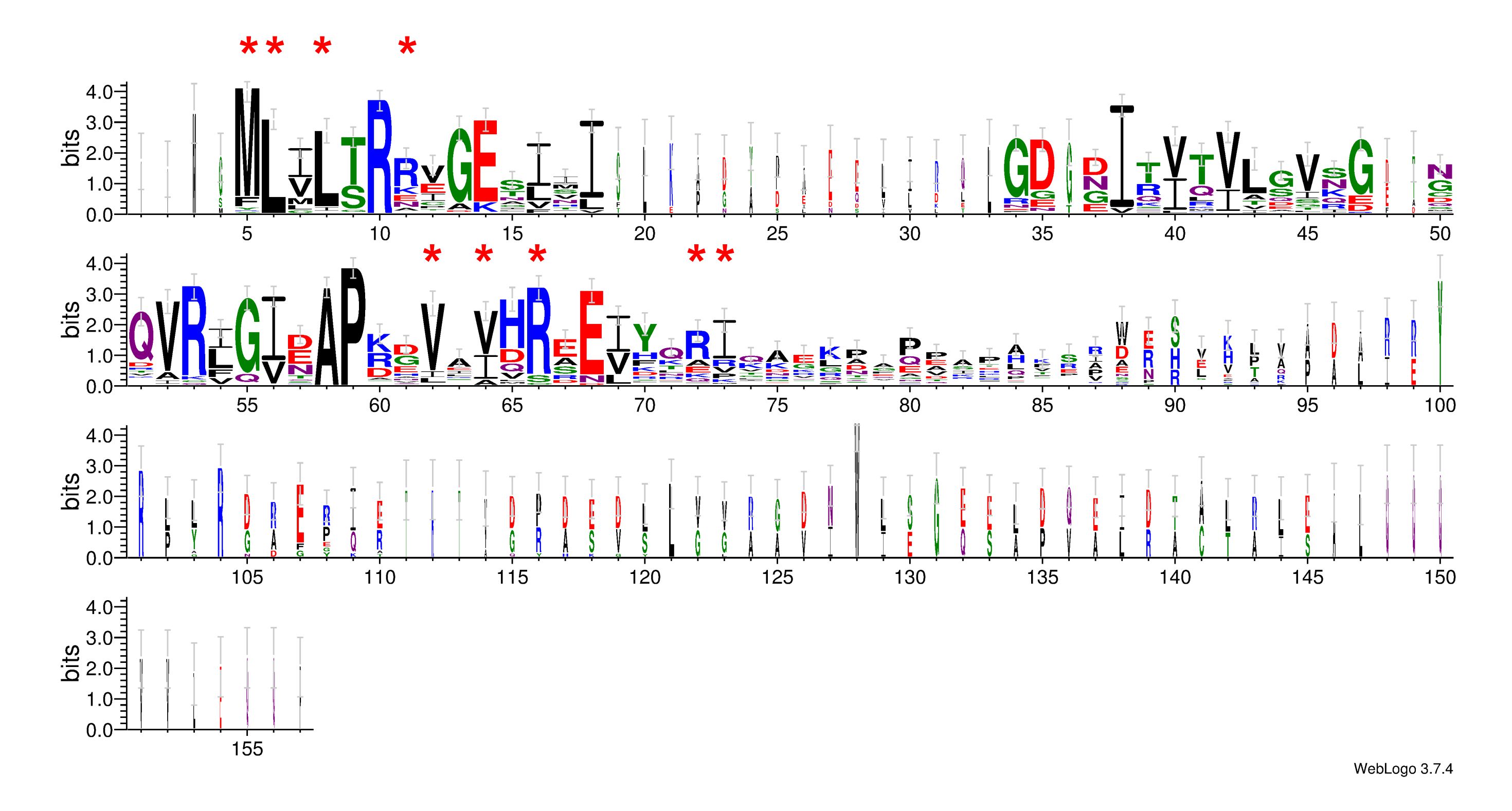

Supplement: Supplementary Figure 3 — Amino acid conservation across all families of Pseudomonas Rsm paralogues. A multiple alignment was generated with MUSCLE (Edgar, 2004) containing all the 45 Rsm paralogues used for the phylogenetical inference (see Figure 6). The sequence LOGO is the graphical representation of this multiple alignment. [file Image_3.JPEG]

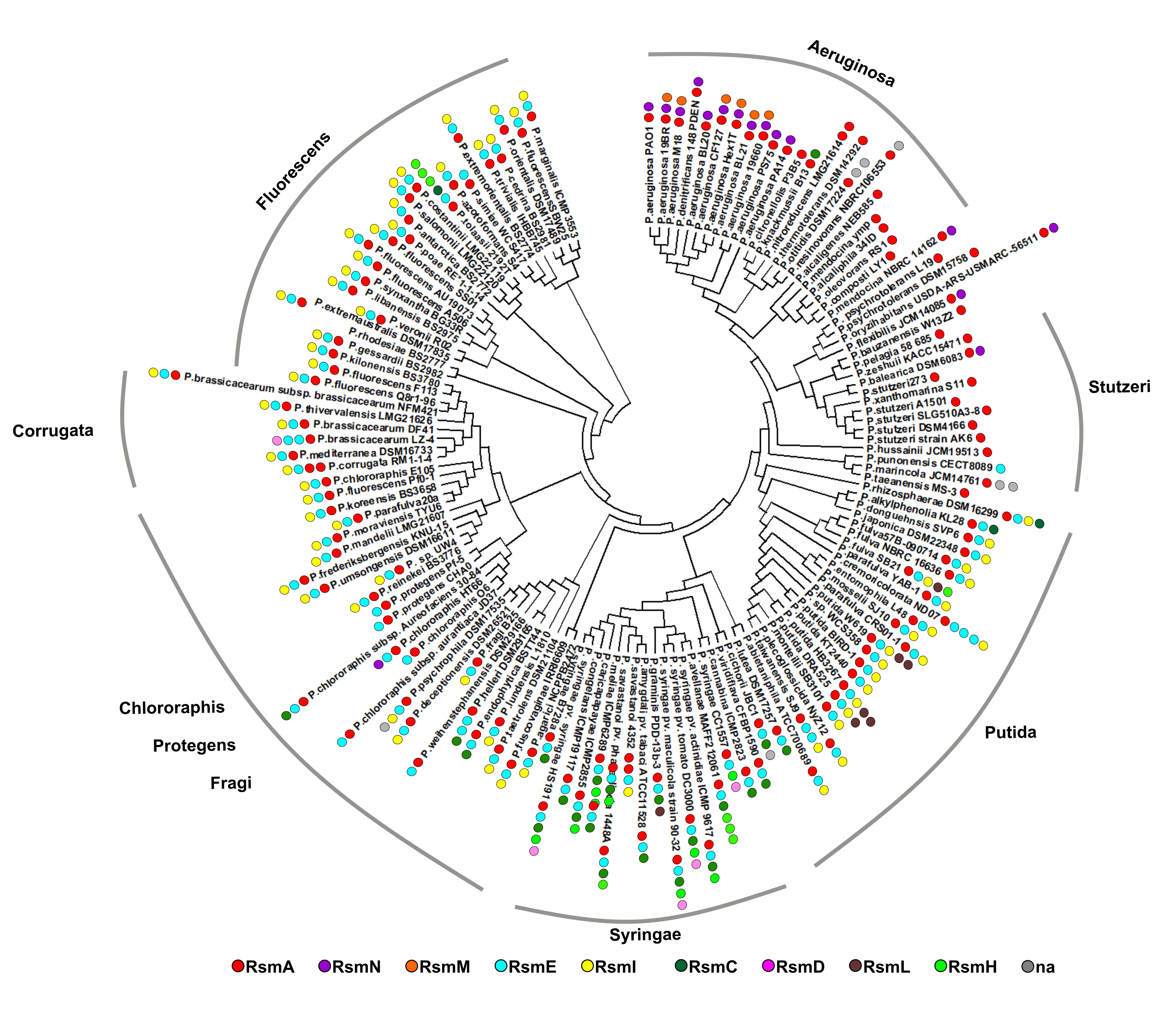

Supplement: Supplementary Figure 4 — Phylogenetic distribution of members of the Rsm paralogue subfamilies within the genus Pseudomonas. The phylogeny of the genus was built using the sequence of the gyrB gene. The evolutionary history was inferred using the Neighbor-Joining method (Saitou and Nei, 1987). The optimal tree with the sum of branch length = 5.65 is shown. The evolutionary distances were computed using the Kimura 2-parameter method (Kimura, 1980) and are in units of number of base substitutions per site. This analysis involved 138 nucleotide sequences. All positions with <95% site coverage were eliminated, i.e., fewer than 5% alignment gaps, missing data, and ambiguous bases were allowed at any position (partial deletion option). There were a total of 2,414 positions in the final dataset. Evolutionary analyses were conducted in MEGA X (Kumar et al., 2018). For each Pseudomonas strain, the subfamily of Rsm paralogues present in the corresponding genome is indicated with colored filled circles, following the color scheme shown at the bottom of the figure. [file Image_4.JPEG]
